# Interpreting higher-order dependence in multimorbidity using cohort data: A partial information decomposition approach

**DOI:** 10.1371/journal.pcbi.1014386

**Published:** 2026-06-10

**Authors:** Cillian Hourican, Geeske Peeters, René J. F. Melis, Almar Kok, Natasja M. van Schoor, Sandra Wezeman, Mike Lees, Marcel G. M. Olde Rikkert, Rick Quax

**Affiliations:** 1 Computational Science Lab, Informatics Institute, University of Amsterdam, Amsterdam, The Netherlands; 2 Department of Geriatric Medicine, Radboud University Medical Center, Nijmegen, The Netherlands; 3 Radboudumc Alzheimer Centre, Radboud University Medical Centre, Nijmegen, The Netherlands; 4 Amsterdam UMC location Vrije Universiteit Amsterdam, Department of Epidemiology and Data Science, Amsterdam, The Netherlands; 5 Department of Psychiatry, Amsterdam Public Health Research Institute, Amsterdam University Medical Centers, Amsterdam, The Netherlands; 6 Amsterdam Public Health Research Institute, Aging and Later Life, Amsterdam, The Netherlands; 7 Institute for Advanced Study, Amsterdam, The Netherlands; University of Colorado Anschutz Medical Campus, UNITED STATES OF AMERICA

## Abstract

In the context of multimorbidity, clinical features seldom act in isolation: symptoms, signs and behaviours form interdependent systems in which joint effects on function can be demonstrated only when features are considered together. We introduce an open, reusable workflow that detects and interprets these “together-only” interactions using bivariate Partial Information Decomposition (PID; two sources to one target), linking synergy-based dependence to the broader network of clinical variables rather than to a single target. The workflow estimates synergy with small-sample bias correction and summarises each pair in a Breadth–Uniformity–Synergy–Total (BUST) map: *breadth* of synergy across *target variables* (broad “generalist” vs narrow “specialist” patterns), cross-stratum *uniformity* across age, sex and multimorbidity (uniform vs subgroup-specific), synergy strength, and total shared information. Simple diagnostics contrast observed targets with additive expectations, revealing the specific joint configurations through which non-additive effects arise. Applied to data from the Longitudinal Ageing Study Amsterdam, we treated all health-related variables—covering symptoms, clinical signs, behaviours, lifestyle factors, and self-rated health indicators—as both sources and targets in the PID framework. This symmetric design permits synergy to be quantified for every pair of variables with respect to every other variable. The workflow identifies synergistic constellations that additive models miss. Multidomain cliques involving subjective health, pain, cognition and grip strength showed multiple non-additive configurations, whereas pairs such as alcohol use with grip strength exhibited focused, narrow but uniform synergy. Notably, the pairs with the strongest synergistic contributions were largely distinct from those with the highest total mutual information, indicating that synergy captures dependency structure overlooked by conventional association measures. Rather than a new measure, this work provides a bias-aware workflow that makes higher-order dependence visible and transferable. Our results support synergy-aware mapping as a practical complement to conventional multimorbidity analyses: it highlights specific combinations of routinely assessed features whose joint states may be especially informative across multiple health targets and therefore candidates for prioritised joint assessment and future multi-domain intervention studies.

## Author summary

When older adults live with multiple health conditions simultaneously - a situation called multimorbidity - their symptoms and health indicators can interact in surprising ways. Two variables that seem unremarkable on their own may together carry important predictive information about a third. Standard statistical approaches, which typically examine variables in pairs, cannot detect these joint effects. We apply a method from information theory called Partial Information Decomposition to systematically identify such “synergistic” pairs across a large set of health variables measured in a Dutch cohort of older adults. We then introduce the BUST framework (Breadth, Uniformity, Synergy, Total) to classify these pairs according to how many outcomes they jointly predict, how consistently they appear across demographic subgroups, and how strong their effects are. We find that the most synergistic pairs are entirely different from those with the strongest overall statistical associations, meaning conventional analyses would miss them. Sleep quality, physical activity, self-rated health, depressive symptoms, and grip strength emerge as central variables in these synergistic interactions. Our open-source pipeline can be applied to other cohort datasets, offering researchers a new lens for understanding how health variables combine in complex disease.

## 1 Introduction

Clinical features seldom act alone. In high-dimensional clinical data, multiple measurements can carry information about targets that is not present in any single variable. This is referred to as *synergy*: information about a target that emerges only when two features are observed together. Detecting such interactions is difficult for several reasons: The number of candidate feature sets grows combinatorially; multivariate information estimates can be biased in finite samples; and higher-order effects are challenging to interpret because many analytical tools either blur pair-level specificity or produce triplet-level results that are hard to compare across targets and subgroups. An interpretation-first approach is therefore needed if higher-order dependence is to inform clinical reasoning and study design [1,2].

This need is particularly evident in multimorbidity, where co-occurring symptoms and conditions lead to heterogeneous functional trajectories. Conventional multimorbidity approaches—disease counts and composite indices; regression with main effects and occasional interaction terms; clustering or latent classes; and pairwise networks—characterise co-occurrence and additive burden but offer limited traction on genuinely irreducible joint influences. For example, network- and cluster-based analyses in large cohorts have identified reproducible constellations of cardiometabolic, musculoskeletal and mental-health conditions, but these remain rooted in co-occurrence patterns rather than in how diseases and symptoms jointly influence function or other targets [3,4].

These conventional approaches also blur two distinct notions of “interaction”. In epidemiology, interaction typically refers to effect modification on a chosen scale (e.g., additivity or multiplicativity) within a specified model. Here we use an information-theoretic notion: two variables exhibit *synergy* for a target when observing them jointly reduces uncertainty about that target more than would be expected from their individual contributions alone. Because this definition is model-free, synergy need not align with a particular regression interaction term or link function. This distinction matters: symptom burden often outperforms disease counts for predicting function, and certain constellations behave ”worse than the sum of their parts” [5–8]. Similar principles apply in treatment, where combined management of pain, depression, insomnia or other multimorbidity-related conditions can be more effective than isolated interventions [9–12]. What remains unclear is how to identify, *a priori*, which combinations of clinical features are likely to interact non-additively.

Information theory provides a model-free framework to address this gap. In bivariate Partial Information Decomposition (PID), the information that two sources carry about a target is partitioned into *unique*, *redundant*, and *synergistic* components, enabling direct assessment of whether paired observations convey more information jointly than individually. Multivariate extensions such as the O-information capture redundancy–synergy balance at larger scales and have revealed synergy-dominated subsystems in biological and neural data [1,13–16]. However, two barriers have limited widespread application in epidemiologic and cohort settings. First, detection is fragile: multivariate estimators exhibit small-sample bias (i.e., can be inaccurate when we have insufficient data), and naïve pipelines can misclassify redundancy as synergy or vice versa [1]. Second, interpretation is underdeveloped: triplet-level results are hard to compare across many targets or population subgroups, and existing summaries either remain global (losing pair-level specificity) or hyper-local (difficult to generalise).

In this paper, we propose a bias-aware, interpretation-first workflow that addresses these limitations. The workflow couples permutation-screened PID estimation with a pair-centric summary of how synergy is organised across targets and populations. To make the results clinically legible, we summarise each pair’s behaviour using a Breadth–Uniformity–Synergy–Total (BUST) representation. *Breadth* quantifies how widely a pair’s synergy is distributed across targets (broad vs narrow), whereas *Uniformity* quantifies how consistently these effects appear across age, multimorbidity and sex strata (universal vs specific). *Synergy* and *Total information* capture the magnitude and context of these effects. The BUST map collapses many triplets into a smaller set of patterns that are easier to interpret—for example, pairs whose synergy is broad and consistent across the population, pairs with narrow but reliable effects, and pairs whose behaviour varies across demographic or multimorbidity strata. To connect these information-theoretic quantities to familiar statistical concepts, we also compare observed prediction surfaces against additive and multiplicative baselines for representative triplets (Section S3 of S1 Appendix).

As a use case, we apply the workflow to data from the Longitudinal Ageing Study Amsterdam (LASA) [17]. Because multimorbidity in older adults is often experienced as symptom burden and functional limitation rather than as a list of diagnoses, we focus on routinely assessed symptoms, clinical signs, behaviours and lifestyle factors, self-rated health indicators, and cognitive and sensory measures. Chronic disease information was used only to define multimorbidity strata and was not itself treated as a source or target variable in the PID analysis. With the exception of stratification variables (age, sex, and multimorbidity), all health-related variables were treated symmetrically as both sources and targets: each variable could appear either in a source pair or as a target in a source–source–target triplet. We repeat analyses within age, sex and multimorbidity strata to characterise non-uniformity across subgroups.

The overall aim of this paper is to introduce and demonstrate a structured, bias-aware workflow for detecting and interpreting higher-order informational interactions between clinical features in cohort data, in a way that supports multimorbidity research. Our aims are fourfold. First, we quantify the prevalence and magnitude of triplet-level synergy in this cohort. Second, we identify which variable pairs inform broadly versus narrowly across targets and assess how consistently these patterns are expressed across demographic and multimorbidity strata. Third, we test whether synergistic information is diffuse across many pairs or concentrated in a few. Fourth, we evaluate how synergy relates to more traditional dependence measures such as correlation and total mutual information (TMI). Prior evidence suggests that synergy and strong TMI often cluster in constellations involving sleep, pain, anthropometry and grip strength, while several disease–disease pairs act more broadly with moderate magnitude [6–8]. Our contribution is a bias-aware PID workflow and a Breadth–Uniformity–Synergy–Total (BUST) representation that render these patterns interpretable at the level of individual pairs.

This design preserves clinical relevance because the measured variables span domains central to independent living and routinely assessed geriatric evaluation, while the pair-centric summaries mirror how clinicians reason about co-present problems [5].

In summary, this study contributes a reproducible workflow for detecting and debiasing higher-order information in cohort data and an interpretation-centred BUST representation that makes higher-order dependence clinically legible by design. Applied to LASA, the workflow maps where synergistic information resides in the data and how broad and uniform the synergies are. Although the analyses are observational and do not imply causality, this workflow is domain-agnostic and directly transferable to other biomedical settings in which complex constellations, rather than isolated predictors, may drive outcomes [1,2,13,14].

## 2 Results

### 2.1 Prevalence and magnitude of synergistic information

Before decomposing shared information into synergistic, unique and redundant components, we first examined how often pairs of variables jointly carried any information about a third variable. With *V* = 17 analysed variables treated symmetrically as potential targets, this yields V×(V−12)=17×120=2,040 distinct variable-pair–target triplets per dataset (i.e., all pairs among the remaining variables for each target). Across all age, sex and multimorbidity strata, 86–97% of these two-predictor–one-target triplets showed statistically significant joint mutual information under permutation testing with false-discovery-rate (FDR) control. In the full sample, 2,005 of 2,040 tested triplets (98.3%) had significant joint mutual information (FDR thresholds 0.042–0.048; Table A in S1 Appendix). These results indicate that joint dependence between variables is therefore widespread. We then examined how much of this shared information is truly *synergistic*, as opposed to being explained by redundant or unique contributions.

**Fig 1:**
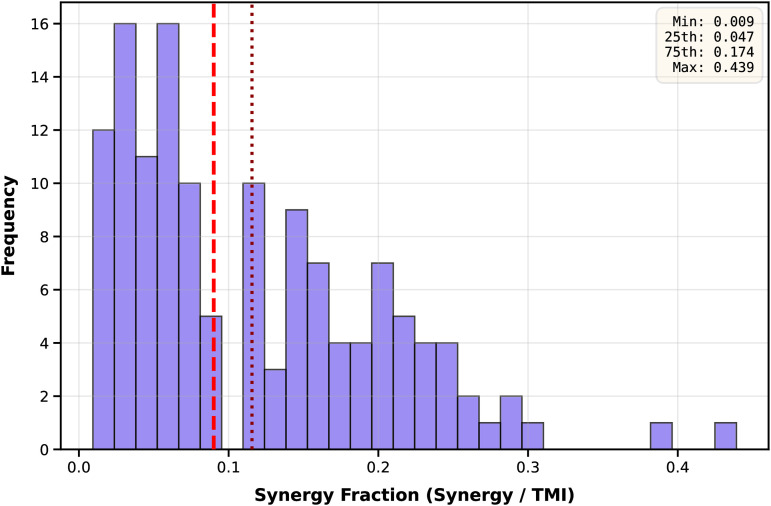
Synergistic information constitutes a small but heterogeneous fraction of total mutual information (TMI). Distribution of synergy fraction (synergy strength / TMI) across 135 source pairs. The right-skewed distribution indicates that a minority of relationships exhibit disproportionately strong synergistic contributions. The vertical dotted lines show the median (long dashes) and mean (short dashes).

Across the 135 synergistic source pairs (pairs that have statistically significant synergy for at least one target), synergy constituted a small but widely varying fraction of TMI (Fig 1). The distribution of synergy fractions was right-skewed, with a median of 0.090; the interquartile range was 0.047–0.174. This indicates that, for most pairs, fewer than 10% of shared information bits arose from joint effects, whereas a minority of pairs exhibited substantially larger fractions. Synergistic effects are therefore selective rather than ubiquitous, motivating methods that explicitly identify these rare but informative interactions.

To assess the stability of these estimates, we computed bootstrap confidence intervals (1,000 replicates). Across the top-10 BUST pairs, 95% CIs for mean synergy were narrow (median width 0.005 bits) and all excluded zero (Fig L in S1 Appendix). At the individual-target level, 73–93% of targets per pair had synergy CIs that excluded zero, indicating that the synergistic signal is not driven by a small subset of targets.

### 2.2 Simulation validation

Three simulation experiments confirmed that the QE bias-corrected pipeline recovers known PID atoms and BUST scores under conditions matched to the empirical data (Methods Section 4.6; Section S5 of S1 Appendix). In the cardinality sweep (128 conditions, 50 replicates each), estimated PID atoms matched ground truths with mean absolute bias below 0.01 bits and RMSE below 0.05 bits for *K* ≤ 4, with 95% confidence interval coverage between 90% and 98%. At *K* = 5 with *N* = 500 a small positive synergy bias (≈0.035 bits) appeared but diminished at larger sample sizes. Under LASA-matched asymmetric cardinality profiles, all atoms were recovered with RMSE < 0.02 bits at *N* = 2,636, and regimes with zero true synergy produced negligible estimated synergy (bias < 0.001 bits at N≥1,000). BUST scores were also faithfully recovered: synergy strength and total information had RMSE < 0.13 and < 0.008 bits respectively, and the relative ranking of pairs by all three BUST components was preserved. Breadth exhibited a conservative downward bias for broad-synergy pairs, meaning pairs classified as “broad” in the empirical BUST map genuinely possess broad profiles.

### 2.3 Breadth and uniformity of synergy across targets and strata

**Fig 2:**
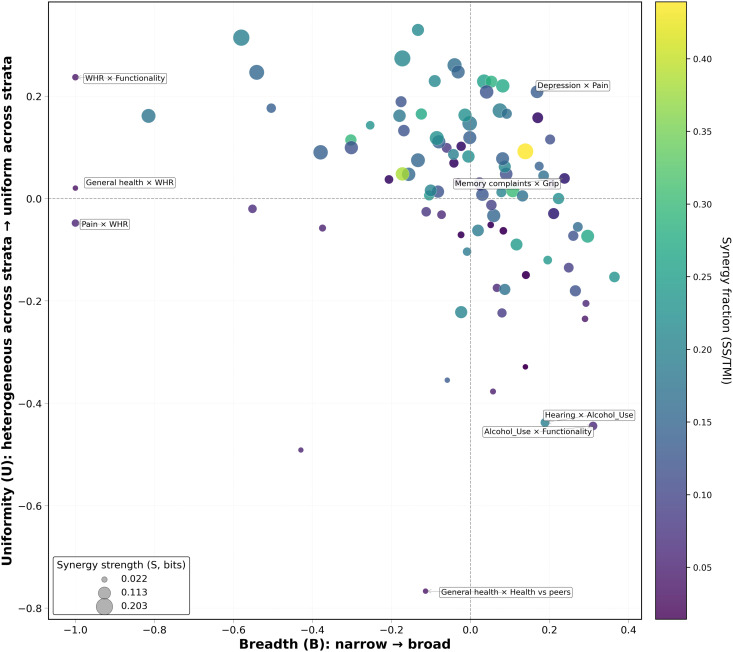
BUST map summarises synergistic source pairs by breadth and uniformity. Each point represents one source pair (*n* = 135). The *x*-axis is Breadth (*B*), measuring how widely a pair’s synergy is distributed across targets; the *y*-axis is Uniformity (*U*), measuring how consistently that synergy appears across age, sex, and multimorbidity strata (see Methods). Both axes are median-centred, so *B* = 0 and *U* = 0 correspond to a typical pair; dashed lines at zero define four archetypes: broad–uniform (BU), narrow–uniform (NU), broad–specific (BS), and narrow–specific (NS). Point size encodes synergy strength (bits) and colour encodes TMI (bits). The two most extreme pairs are annotated in each quadrant.

After estimating synergy for each source pair across all targets and strata, we summarised each pair on the BUST map (Fig 2). Breadth (*B*) quantifies how widely a pair’s synergistic information is distributed across targets, whereas Uniformity (*U*) quantifies how consistently that synergy appears across age, sex, and multimorbidity strata. Both scores are median-centred, so *B* = 0 and *U* = 0 represent a “typical” pair in this dataset. Pairs with *B* > 0 exhibit broader-than-typical synergy profiles (spread across more targets), whereas *B* < 0 indicates synergy concentrated on a smaller subset of targets. Similarly, *U* > 0 indicates more uniform-than-typical synergy across strata, whereas *U* < 0 indicates subpopulation-specificity (stronger variation across strata).

The highest composite BUST score (min–max normalised to [0,1] across pairs) was observed for perceived health relative to peers (i.e., one’s own health rating compared to the estimated health of peers) with physical activity (composite = 1.000), which combined strong synergy (0.20 bits) with positive Uniformity (U = 0.27) and slightly below-median Breadth (B = -0.17). This indicates a synergy pattern that is expressed consistently across strata but is concentrated on a subset of targets. Other top-ranked pairs included alcohol use with grip strength (BU; composite = 0.945), pain symptoms with physical activity (NU; composite = 0.915; the highest strength at 0.20 bits), sleep quality with perceived health relative to peers, depression with perceived health relative to peers, sleep with physical activity, cognition with (perceived) general health, general health with grip strength, BMI with alcohol use, and sleep with alcohol use. These interactions form an interpretable pattern in which subjective comparative health, sleep, pain, mood, activity, body composition, cognition, and physical capacity jointly encode non-additive information about multiple targets, including functional limitations.

Composite BUST scores provide a compact entry point by ranking source pairs that jointly combine (i) non-trivial synergy strength, (ii) above-typical Breadth across targets, and (iii) above-typical Uniformity across strata (Methods Section 4.4). Importantly, BUST does not reduce each pair to a single number: it retains each pair’s Breadth and stratum-specific Uniformity components, which enables targeted follow-up views once the full set of BUST scores has been computed. We therefore complement the composite ranking with an explicit analysis of low-uniformity pairs, which isolates interactions whose synergy profiles vary most across age group, sex, or multimorbidity status.

#### Context-dependent synergies and drivers of non-uniformity.

**Fig 3:**
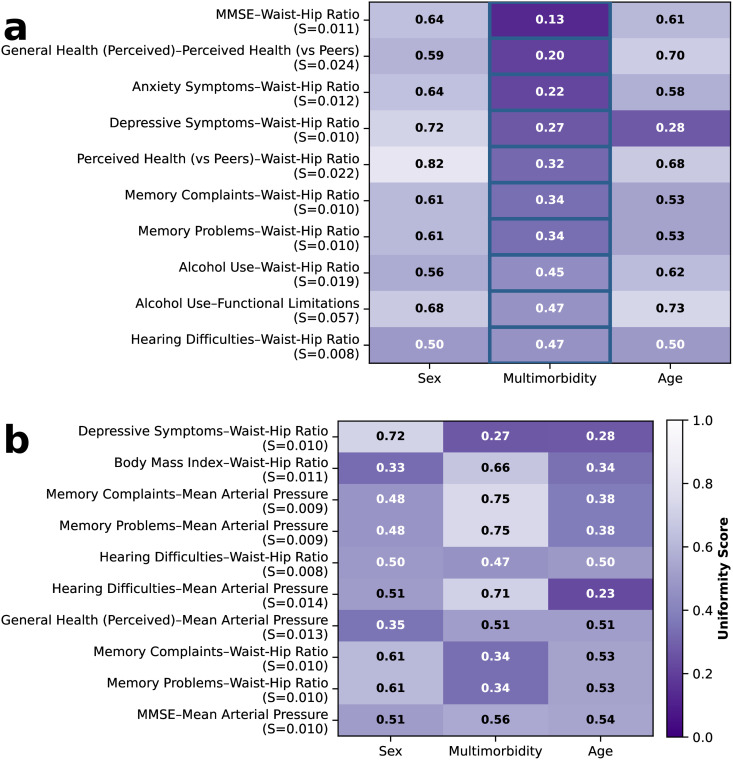
Low-uniformity pairs reveal which stratification drives context-dependent synergy. **(a)** Pairs with the lowest multimorbidity-specific uniformity component. **(b)** Pairs with the lowest median uniformity across sex, multimorbidity, and age. Heatmaps show the entropy-based uniformity components for each source pair across sex, multimorbidity status (present vs. absent), and age group. Lower values indicate greater between-stratum variability in the pair’s synergy profile for that stratification, whereas higher values indicate more stable behaviour across strata. Row labels report each source pair and its synergy strength *S* (bits).

Uniformity in the BUST representation is computed separately for each stratification variable (sex, multimorbidity status, and age group) and then aggregated for the 2D map. To make the “specific” (low-uniformity) region of the map interpretable in practice, we ranked source pairs by their *stratum-specific* uniformity components and visualised the lowest-uniformity examples (Fig 3). Each row shows a source pair, and each column reports the entropy-based uniformity score for that stratification, where lower values indicate greater between-stratum variation in the pair’s synergy profile.

Fig 3a focuses on multimorbidity status (present vs. absent) by showing the ten pairs with the lowest multimorbidity uniformity component. For these pairs, the multimorbidity column is systematically lower than the corresponding sex- and age-based uniformity components, indicating that their context dependence is driven primarily by differences between the multimorbidity strata rather than by sex or age.

Fig 3b provides a complementary, global view by selecting the ten pairs with the lowest *median* uniformity across sex, multimorbidity, and age. In this view, low overall uniformity arises for different reasons: for some pairs the lowest component is age-based, for others it is sex-based, and for others it is multimorbidity-based. This decomposition shows that “non-uniform” synergy is not a single phenomenon: the same overall label can reflect instability along different subgroup dimensions, which guides which stratified analyses are most relevant for follow-up.

### 2.4 System-level organisation of synergy

**Fig 4:**
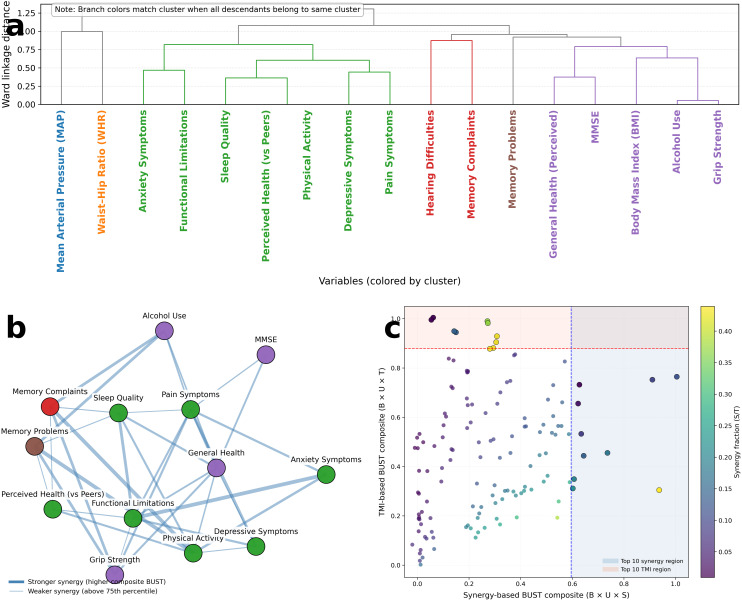
Hierarchical, network, and ranking perspectives on synergistic structure under the BUST representation. **(a)** Dendrogram from Ward hierarchical clustering on a distance matrix derived from composite BUST edge weights. Coloured branches indicate clusters identified below a threshold of 0.7× the maximum linkage distance; grey branches represent inter-cluster connections. **(b)** Weighted BUST network retaining only edges above the 75th percentile of the composite weight distribution; isolated nodes are omitted. Node colours follow dendrogram cluster assignments. Edge thickness is scaled nonlinearly with the composite weight. Absence of an edge or node does not imply independence, but indicates that the pair’s composite weight fell below the plotting threshold. **(c)** Composite rankings using synergy strength as the magnitude term (BUS, *x*-axis) versus total mutual information (BUT, *y*-axis), with Breadth (*B*) and Uniformity (*U*) held fixed. Dashed lines mark the top-10 cutoff under each composite; shaded regions identify pairs ranking in the top 10 under each definition. Point size encodes synergy strength (*S*, in bits); colour encodes synergy fraction (*S*/TMI).

We then examined how these pairwise synergies are organised at the system level. Using composite BUST scores as edge weights, we constructed a weighted synergy network in which each variable is connected to every other variable. Despite this near-complete connectivity, hierarchical clustering of the associated distance matrix (1 minus the composite BUST weight) revealed a structured organisation consisting of four groups (Fig 4a). Waist–hip ratio (WHR) and blood pressure (mean arterial pressure, MAP) formed small metabolic and cardiovascular branches, respectively. A larger subjective-health and functioning cluster grouped anxiety symptoms, perceived health relative to peers, pain symptoms, physical activity, sleep quality and functional limitations. The remaining variables formed a cognition/comorbidity cluster linking alcohol use, BMI, depression, general health, hearing difficulties, cognition, memory complaints, memory problems and grip strength. These groupings align with established geriatric syndrome domains (i.e., malnutrion; cardiovascular ageing; psychosocial functioning; dementia, sarcopenia), suggesting that synergistic information recovers clinically meaningful higher-order structure.

To make the multivariate organisation visually explicit, we plotted a thresholded BUST network in which nodes represent variables and edges represent composite BUST scores (Fig 4b). For clarity, we retained only edges above the 75th percentile of the composite distribution (34 of 135 possible edges) and removed nodes with no such edges. This representation reveals a compact backbone centred on sleep quality, pain symptoms, perceived health relative to peers, physical activity, depression, grip strength and alcohol use.

#### 2.4.1 Stable synergistic constellations.

Finally, we identified small groups of variables whose pairwise synergies remain consistently strong across multiple thresholds. Starting from the weighted BUST network, we created four unweighted graphs by retaining edges with composite BUST scores above the 67th, 75th, 80th and 85th percentiles. A clique was defined as stable if it appeared in at least three of these four thresholded graphs, highlighting local constellations whose pairwise synergies remain strong across a range of thresholds.

Eighteen stable maximal cliques were found, comprising sixteen triplets and two quadruplets. One recurrent multidomain constellation linked general health, pain symptoms, grip strength and cognition. This clique exhibited comparatively strong and widespread synergies across multiple targets. Joint-state analyses within this clique revealed several configurations that deviated markedly from additive expectations for targets including sleep, physical activity, hearing, memory complaints and functional limitations, and rule-augmented models frequently improved model fit (Section S4 of S1 Appendix). Clique members differed in how many joint configurations exhibited improved fit relative to additive expectations: some cliques were dominated by a single configuration (e.g., alcohol use–grip strength), whereas others involved multiple configurations (e.g., general health–pain–grip strength–cognition)

A second stable quadruplet—alcohol use, general health, pain symptoms and grip strength—shared three variables with the first but showed a more focused configuration structure. The alcohol use–grip strength pair was among the strongest BUST edges overall and had one of the highest synergy strengths. For each target examined, a single joint state drove the largest deviations from the additive model, yet this configuration was sufficient to improve fit for several targets, including depression, general health, perceived health relative to peers and memory problems (Section S4 of S1 Appendix). This behaviour is consistent with a sharp, high-impact interaction nested within an otherwise broader synergy pattern.

Among triplets, the clique comprising perceived health relative to peers, physical activity and sleep quality had the highest mean composite BUST score and was present at all thresholds. The sleep–physical activity edge within this set was one of the strongest composite BUST edges in the network and also featured prominently in the BUST map. Joint-state analyses indicated that this behavioural clique occupies an intermediate position between the two quadruplets, with multiple abnormal configurations and model-fit improvements concentrated in pain, grip strength and functional targets (Section S4 of S1 Appendix).

### 2.5 Relationship between synergy and conventional dependence measures

We evaluated how PID-based synergy compares with traditional dependence measures such as TMI. If strong synergy simply reflected high overall information sharing, then ranking pairs by TMI would recover the same source pairs as ranking by synergy. We therefore constructed two composite scores that summarised each pair’s breadth and cross-stratum consistency but differed in their magnitude component: one used synergy strength and the other used TMI (Methods Section 4.4).

Synergy strength and TMI were only weakly correlated across the 135 source pairs (Pearson *r* = 0.31, *p* < 0.001), and the top-10 pairs under the synergy-based composite showed *zero overlap* with the top-10 pairs under the TMI-based composite (Fig 4c). Several high-TMI pairs, such as combinations of memory complaints and problems, had modest synergy fractions, whereas strongly synergistic pairs—such as alcohol use–grip strength, sleep–physical activity and perceived health relative to peers–physical activity—occupied mid-range TMI values. This confirms that synergy-based rankings are not recoverable from TMI alone and underscores the added value of PID relative to correlation or TMI screening. Several TMI-composite top pairs were tied, consistent with near-redundant variables yielding similar total-information profiles.

## 3 Discussion

In this study, we introduced an interpretation-first pipeline that couples bias-corrected partial information decomposition with pair-centred summarisation via a Breadth–Uniformity–Synergy–Total (BUST) representation. Applied to multimorbidity-related data from LASA, this approach mapped how synergistic information is distributed across symptoms, signs, and other health-related variables across a broad set of health and functional targets. The main findings are that: (i) synergy represents a small varying fraction of total shared information across triplets; (ii) relationships ranked highest by synergy are entirely distinct from those ranked highest by total mutual information (TMI); (iii) synergistic pairs occupy distinct regions of the BUST map that differentiate broad and uniform synergies from narrow or context-dependent (specific) ones; (iv) robust multi-way synergies concentrate in small, cross-domain cliques; and (v) the resulting synergy network is dense yet hierarchically organised, with different variables acting as integration hubs versus structural bridges.

More broadly, the BUST representation aligns with symptom-oriented views of multimorbidity in which patient experience and functional status emerge from interacting problems across domains rather than from diagnoses considered in isolation. By mapping which symptom/sign/behaviour pairs carry irreducibly joint information across targets and strata, BUST complements diagnosis-based multimorbidity phenotyping and may help prioritise cross-domain constellations for assessment and hypothesis generation.

### 3.1 PID reveals information architecture beyond mutual information

The complete non-overlap between the top-10 synergy-based and top-10 TMI-based composite rankings demonstrates that bivariate PID provides non-redundant information relative to conventional mutual information analysis. High TMI pairs were not simply high-synergy pairs with additional redundant content; instead, they belonged to a distinct set of relationships whose shared information was dominated by redundant and unique components. Conversely, strongly synergistic pairs occupied mid-range TMI values and were not identifiable by total information content alone.

### 3.2 BUST archetypes recover expected constellations and highlight new candidates

The BUST map places individual source pairs into archetypes and helps link synergistic patterns to clinical narratives (Fig 2). Several of the strongest and most clinically interpretable pairs (i.e., pairs with plausible joint-state narratives in routinely assessed domains) fall in the broad–uniform (BU) and narrow–uniform (NU) quadrants, reflecting synergy that is expressed consistently across age, sex and multimorbidity strata. Examples include sleep with physical activity, sleep with perceived health relative to peers, alcohol use with grip strength, and depression with perceived health relative to peers. These pairs combine positive Uniformity with either broad or narrower Breadth and jointly encode information about many functional outcomes. Their positions on the BUST map are consistent with evidence that poor sleep, low physical activity, depression, problematic alcohol use and perceived poor health tend to cluster within individuals and are collectively associated with declines in physical and mental health. For instance, depression and excess weight have been shown to reinforce each other bidirectionally in longitudinal cohorts [18,19], and such mood–weight constellations often coexist with disturbed sleep and low activity in older adults. Perceived health relative to peers is particularly notable: self-rated health integrates information from multiple domains and predicts retirement due to disability and mortality beyond objective diagnoses [20,21]. Its repeated appearance in high-scoring BU and NU pairs suggests a role as an integrative “health signal” that synergises with behavioural and mood-related variables.

Pairs in the broad–specific (BS) quadrant exhibit synergy that spans multiple targets but varies across strata. These include sleep with alcohol use, pain with alcohol use and sleep with hearing difficulties. Their placement is consistent with evidence that alcohol-related effects on metabolic and behavioural functioning differ substantially across intake levels and population subgroups. Heavy drinking is more strongly linked with adiposity and cardiometabolic risk, whereas light or moderate consumption shows weaker or mixed associations [22,23]. Similar variation is reflected in population studies reporting associations between alcohol intake, abdominal obesity and health-related behaviours [24]. The BS quadrant, therefore, captures pairs whose joint behaviour is broad but modulated by demographic or multimorbidity context.

Pairs in the narrow–specific (NS) quadrant exemplify concentrated but population-dependent synergies. These include cognition with perceived health relative to peers and several combinations involving BMI, waist–hip ratio (WHR), or blood pressure. In these cases, synergy is consistent with subgroup-conditional coupling between subjective appraisal and objective markers: combinations of self-rated health with cognitive or physical capacity may carry joint information that is not captured by additive contributions alone, and the strength of this coupling can vary across strata. Similarly, configurations combining functional limitations with poorer perceived health reflect closely related constructs, but the presence of synergy suggests that their joint states can still encode non-additive information about other targets, in a way that differs across subgroups.

Overall, these quadrant-level patterns serve two purposes. First, they provide face validity by showing that BUST recovers clinically familiar constellations—such as links among sleep, perceived health, mood, physical activity, and alcohol use—in positions that match established knowledge. Second, the map highlights pairs whose interactions are less well characterised in routine multimorbidity analyses, including pain–alcohol use, anxiety–physical activity and sleep–alcohol use. These combinations are not typically examined together despite plausible mechanistic connections, and their location on the BUST map suggests synergistic behaviour that is strong yet not readily apparent from correlation or co-occurrence alone. Such pairs, therefore, represent promising candidates for deeper mechanistic or interventional study. This heterogeneity suggests that synergy-informed applications may require both population-wide constellations (e.g., alcohol use–grip strength, sleep–physical activity) and more targeted, domain-focused interactions (e.g., pain–physical activity, perceived health relative to peers–physical activity).

Low-uniformity pairs add a clinically relevant layer to this picture. A pair can show clear synergistic behaviour in the pooled sample while still behaving differently between subgroups, meaning that a single population-wide summary may not be appropriate for screening, risk stratification, or hypothesis generation. Decomposing uniformity into its sex-, age-, and multimorbidity-specific components helps distinguish interactions that are broadly stable from those whose joint implications differ between participants with versus without multimorbidity or across age groups. One interpretation is that multimorbidity status can change the context in which symptoms, behaviours, and physiological markers co-occur, so that the same two-variable constellation may encode different joint information depending on baseline health complexity. In practice, this argues for reporting not only a single BUST position but also which stratification dimension drives any observed context dependence when translating synergistic relationships to clinical assessment or to cohort-to-cohort comparison.

The heterogeneity of BUST archetypes among the strongest pairs also invites interpretation. Several high-ranking narrow–uniform pairs, such as perceived health relative to peers–physical activity, pain–physical activity and depression–perceived health relative to peers, combine relatively focused Breadth with consistent cross-stratum behaviour. These cases can often be understood through clinical mechanisms. For example, the synergy between pain and physical activity may reflect different coping patterns: individuals who remain active despite pain tend to exhibit more adaptive coping and better functional trajectories, whereas those who reduce activity may follow a more passive pattern associated with poorer outcomes. BUST does not distinguish these causal pathways, but it flags that the joint state of pain and activity is more informative about downstream targets than either measure alone. Likewise, narrow–specific constellations such as cognition–perceived general health or cognition–perceived health relative to peers may arise because cognitive performance aligns more directly with an individual’s own perceived health than with more cognitively demanding comparative assessments. These examples illustrate how BUST-derived synergy patterns can map onto clinically meaningful behavioural and cognitive processes, helping to generate mechanistic hypotheses about why certain constellations exhibit strong or population-dependent synergy.

### 3.3 Synergistic clusters mirror established geriatric domains

Our synergy-based clustering also aligned closely with established geriatric domains. The dendrogram separated a metabolic marker (WHR) and a cardiovascular marker (blood pressure) from a cluster of symptoms and behaviours (sleep quality, physical activity, pain symptoms, anxiety symptoms, perceived health relative to peers, and functional limitations), and from a broader cognition/comorbidity cluster (depression, perceived general health, hearing difficulties, cognition, memory complaints and problems, BMI, alcohol use and grip strength) (Fig 4a). This structure is consistent with epidemiological work showing that central obesity and elevated blood pressure are core components of cardiometabolic multimorbidity that predict cardiovascular events and later cognitive decline, effectively bridging metabolic, cardiovascular and neurological pathways [25–28].

Likewise, the close grouping of sleep disturbance, pain, mood and reduced activity echoes symptom-cluster studies in older adults, where co-occurring insomnia, chronic pain and depression are strongly associated with frailty and reduced physical function, and where combinations of such symptoms produce worse outcomes than any single symptom alone [29]. This clinical notion of “worse than the sum of the parts” is closely aligned with the synergistic patterns revealed by the BUST representation, even though the underlying statistical formalisms differ.

These clusters arise purely from the organisation of synergistic information and therefore complement, rather than replicate, additive or correlation-based structures. The cognition/comorbidity cluster, which links depression, hearing loss, subjective memory concerns, poorer perceived general health, and weaker grip strength, also mirrors current understanding of age-related cognitive vulnerability. Hearing impairment and depression have both been associated with increased dementia risk [30], and subjective cognitive complaints are common among patients attending audiology clinics, where multiple dementia risk factors can co-occur [31]. Grip strength and BMI further anchor this cluster in frailty: lower muscle strength and adverse body-composition profiles predict poorer physical and cognitive outcomes in later life [32–34]. Interpreted clinically, the high-weight backbone of synergistic relationships links subjective health, mood, behaviour and physical capacity - domains often jointly implicated in frailty and functional vulnerability - whereas cardiometabolic markers appear to contribute more selectively within this dataset. The emergence of these clinically recognisable constellations from purely synergistic information suggests that the BUST representation captures meaningful higher-order structure in the multimorbidity landscape.

### 3.4 Clinical practice and future directions

From a clinical perspective, our findings suggest that synergy-aware analyses can complement existing multimorbidity tools rather than replace them. Broad, relatively uniform pairs such as sleep with physical activity, perceived health relative to peers with physical activity, and depression with perceived health relative to peers behaved as integrative signals: their joint states carried information about a wide range of functional outcomes and did so consistently across demographic and multimorbidity strata. In practical terms, this supports the use of brief questions on sleep, perceived health, and mood, combined with simple anthropometric and performance measures, as efficient “multi-domain probes” in routine assessment. At the same time, more specific but robust synergies, such as alcohol use with grip strength or BMI, point to particular constellations that may warrant closer scrutiny in individuals who meet specific combinations of behavioural and physical risk factors.

Synergy can be read as a value-of-information signal: it quantifies how much additional predictive content a second measurement contributes given what the first has already revealed. This framing suggests three concrete translation pathways. First, *assessment prioritisation*: in time-constrained settings where comprehensive geriatric assessment is not feasible, BUST-ranked pairs could guide which cross-domain questions to ask together. High-composite pairs such as perceived health relative to peers with physical activity achieved the largest breadth and uniformity scores, suggesting that combining a brief comparative-health question with an activity probe yields more information across multiple targets than either question alone. Second, *context-sensitive follow-up*: when a clinician observes a joint state that belongs to a high-synergy pair - for example, a patient reporting both poor sleep and low physical activity - this combination could prompt targeted screening for outcomes that neither symptom predicts well in isolation (in this case, pain, grip strength, and functional limitations). Third, *subgroup-aware interpretation*: low-uniformity pairs signal that synergistic behaviour differs across strata. For instance, the alcohol use–grip strength pair showed context-dependent behaviour across multimorbidity strata; a clinician might therefore weight this combination differently in a patient with established multimorbidity than in an otherwise healthy individual.

These pathways remain illustrative: translating BUST output into actionable decision rules would require prospective validation, integration with existing risk models, and evaluation of whether synergy-ranked features predict patient outcomes better than simpler alternatives. Because our analyses are observational and based on a single cohort, we view these synergies primarily as hypothesis-generating rather than ready-made decision rules. Future work could use similar pipelines to screen other longitudinal studies, compare BUST patterns across healthcare settings, and embed synergistic pairs into prognostic models or clinical decision support tools. Ultimately, synergy-guided maps of where joint effects concentrate could inform the design of combination interventions - targeting sleep and activity, mood and weight, or alcohol use and physical capacity - that explicitly leverage interactions rather than treating each domain in isolation.

### 3.5 Study limitations

We pooled observations across LASA waves without explicitly modelling within-subject dependence. Waves are spaced approximately three years apart, and many of the variables we analyse (symptoms, signs, wellbeing indicators and functional limitations) change on much shorter time-scales, so repeated measurements are not near-duplicate records and can reflect genuinely different health constellations. Our focus is on population-level dependency structure rather than individual trajectories, and we therefore treat each record as an independent sample from a relatively stable underlying mechanism. Nonetheless, repeated participation introduces some within-person correlation and may lead to a mild underestimation of uncertainty.

Additional limitations relate to study design and measurement structure. The three-year interval between waves constrains temporal resolution, and the available variables may not capture all clinically relevant domains that contribute to synergistic behaviour. Moreover, our analyses rely on discretised versions of predominantly continuous variables, which can inflate or deflate higher-order structure depending on the choice of cutpoints. Continuous PID formulations exist that could, in principle, avoid discretisation altogether. Gaussian PID [35,36] provides closed-form decompositions when all variables are jointly Gaussian, and non-parametric mutual-information estimators based on *k*-nearest-neighbour statistics [37] could serve as building blocks for continuous PID in more general settings. However, our dataset contains an inherent mix of variable types: some predictors are binary (memory complaints, anxiety) or ordinal with few levels (perceived health, depression), while others are originally continuous (grip strength, BMI, blood pressure). Gaussian PID requires all variables to be continuous and jointly Gaussian - assumptions that are violated by binary and coarsely ordinal measures. Methods for PID with mixed discrete–continuous sources remain an open problem in information theory. Our discretisation strategy, while it inevitably discards some fine-grained variation in continuous variables, offers three practical advantages: it handles mixed variable types within a single estimation framework; it produces clinically interpretable categories anchored to established thresholds, and it is fully compatible with the well-characterised $I_{\min}$ redundancy lattice used throughout our analyses. Moreover, the post-hoc sparse-state merging procedure (Section 4.3.1) ensures adequate cell counts for reliable estimation. A binning sensitivity analysis presented in S1 Appendix confirms that the main qualitative findings - including the identity of high-ranking BUST edges and the separation between synergy- and TMI-based rankings - are robust to alternative discretisation choices.

Generalisability is also limited. LASA is a community-based cohort of older Dutch adults, and the synergy patterns observed here may differ in younger populations, in clinical or hospital settings, or in cohorts with different disease burdens or measurement practices. The BUST representation and PID pipeline are directly transferable to such contexts, but the specific locations and magnitudes of synergies should be interpreted as cohort-specific until replicated elsewhere. At the same time, LASA is a population-based cohort sampled from municipal registers across three Dutch regions (Amsterdam, Zwolle and Oss), which supports generalisability of the main patterns to community-dwelling older adults in the Netherlands, even though replication in other cohorts and clinical settings remains necessary.

Despite these limitations, bootstrap resampling (1,000 replicates) confirmed that the top-ranked pairs’ synergy estimates are stable, with narrow 95% confidence intervals that exclude zero for all top-10 BUST pairs (Section S7 of S1 Appendix). Because our conclusions emphasise qualitative patterns - such as the divergence between synergy- and TMI-based rankings and the structure of high-weight BUST edges and cliques - rather than formal inference, the limitations described above are unlikely to alter the main results. Even so, future work could incorporate cluster-aware resampling, subject-level aggregation, or designs with finer temporal granularity to address these considerations more explicitly.

The PID framework generalises naturally beyond two sources. The number of partial information atoms is governed by the Dedekind numbers and grows super-exponentially with the number of sources: from 4 atoms for two sources, to 18 for three, 166 for four, and 7,579 for five [38,39]. While enumerating and estimating these atoms remains computationally feasible for up to approximately five sources, the binding constraint is the joint state space required for reliable estimation. With discrete variables whose levels range from 2 to 7, the number of cells in the joint contingency table grows exponentially: for example, five three-level sources and one three-level target produce 3^6^ = 729 joint states, yielding an average of fewer than four observations per cell in our largest stratum (N≈2,600) - far below the minimum required for stable information-theoretic estimation. Even with four sources the situation is marginal (3^5^ = 243 cells, ∼11 observations per cell). Our focus on bivariate PID (two sources, one target) was therefore a deliberate design choice that balances the ability to detect irreducibly joint effects against the need for adequate statistical power given the sample sizes and variable cardinalities available in the LASA cohort. Future applications to substantially larger cohorts or to variables with fewer discrete levels could extend the analysis to three- or four-source PID and thereby capture higher-order synergies that bivariate decomposition cannot resolve.

### Conclusions

This study introduces a bias-aware, interpretation-focused workflow for detecting and summarising synergistic relationships in cohort data. By combining permutation-screened bivariate PID with the Breadth–Uniformity–Synergy–Total (BUST) representation, network visualisation and clique analysis, we show how higher-order dependence can be mapped in a way that remains interpretable at the level of individual variable pairs. The approach is implemented in open, reusable code and is compatible with standard epidemiological designs, making it directly transferable to other multimorbidity and ageing studies.

Applied to LASA, the BUST representation reveals that synergistic information is present but concentrated: most pairs show small synergy fractions, whereas a minority exhibit clearly “more than the sum of the parts” behaviour. Synergistic pairs occupy distinct regions of the BUST map, with some acting as broad, relatively uniform constellations and others showing narrower or population-specific patterns across age, sex and multimorbidity strata. Relationships ranked highest by synergy are entirely distinct from those ranked highest by TMI, indicating that PID-based synergy captures information architectures that are not recoverable from correlation or TMI alone.

Variables such as sleep, BMI, depression, grip strength and perceived health relative to peers participate disproportionately in high-scoring generalist synergies and act as integrative hubs, while combinations involving alcohol use and physical capacity form more focused but robust specialist synergies. These patterns suggest that a small set of routinely collected measures, including brief questions on comparative health and mood alongside simple functional tests, can yield joint information about daily functioning and related targets that is not available from any single variable alone.

Although our analyses are observational and confined to one cohort, the workflow provides a practical template for future work. Synergy-guided variable maps could be used to refine screening batteries, to prioritise combinations of risk factors for mechanistic study, and to identify candidate constellations for multi-target interventions—for example, jointly addressing sleep and activity, mood and weight, or alcohol use and physical performance. More broadly, the results illustrate that multimorbidity and functional decline are shaped not only by the accumulation of individual deficits but also by how specific symptoms, behaviours and conditions interact, underscoring the value of bringing formal notions of synergy into clinical epidemiology.

## 4 Methods

### Overview of the workflow

We developed an interpretation-first pipeline to detect, debias, and summarise synergistic information at scale. For each target, we (i) screen source–source–target triplets using permutation tests with false-discovery-rate control; (ii) estimate bivariate PID atoms with small-sample bias correction and redundancy-definition sensitivity; and (iii) compress triplet-level results to pair-level profiles via a Breadth–Uniformity–Synergy–Total (BUST) map, which quantifies how widely a pair’s synergy extends across outcomes, how consistent it is across strata, and how strong and information-rich it is overall. Data curation, discretisation, and stratification follow clinical guidance and cohort documentation.

### 4.1 Information-theoretic framework

Mutual information (MI) quantifies the reduction in uncertainty about a target after observing a source. For discrete variables,


$$\begin{array}{c} I(X;Y)=\sum_{x,y}p(x,y)\log\frac{p(x,y)}{p(x)p(y)}\;=\;H(Y)-H(Y|X), \end{array}$$

where H(·) denotes Shannon entropy (log base 2), measured in bits. For two predictors $\left\{X_{1},X_{2}\right\}$, MI does not separate overlap from complementarity. Partial information decomposition (PID) partitions the joint information about *Y* into four non-negative components [38]:


$$\begin{array}{c} I(\{X_{1},X_{2}\};Y)=I_{red}+I_{uniq}(X_{1})+I_{uniq}(X_{2})+I_{syn}, \end{array}$$

interpreted respectively as information shared by both sources (redundancy), information available only from *X*_1_ or only from *X*_2_ (unique), and information that emerges only when both sources are observed together (synergy).

#### Defining the PID atoms.

The decomposition requires a definition of redundancy; all other atoms follow by subtraction. We adopt the $I_{\min}$ measure of Williams and Beer [38], which defines redundancy as the outcome-wise minimum of the specific information each source provides:


$$\begin{array}{c} I_{red}(X_{1},X_{2};Y)=\sum_{y}p(y)\;\min\;\{I_{spec}(y;X_{1}),\;I_{spec}(y;X_{2})\}, \end{array}$$

where the *specific information* of outcome *y* given source *X* is


$$\begin{array}{c} I_{spec}(y;X)=\sum_{x}p(x|y)\;\log\frac{p(y|x)}{p(y)}\;\;\;(\geq 0). \end{array}$$

Intuitively, *I*_red_ captures, for each target outcome separately, the lesser of the two sources’ predictive contributions, then averages over outcomes. The remaining atoms are obtained by subtraction:


$$\begin{array}{c} I_{uniq}(X_{i})=I(X_{i};Y)-I_{red},\;i\in \{1,2\}, \end{array}$$ (1)

$$\begin{array}{c} I_{syn}=I(\{X_{1},X_{2}\};Y)-I(X_{1};Y)-I(X_{2};Y)+I_{red}. \end{array}$$ (2)

#### Alternative redundancy measures.

$I_{\min}$ is a closed-form computation over probability tables and is therefore fast, but it can overestimate redundancy in certain joint distributions [39]. The BROJA measure [39,40] takes a different approach: it defines unique information as the minimum conditional mutual information $I(X_{1};Y|X_{2})$ over all joint distributions *q*(*x*_1_, *x*_2_, *y*) that preserve the observed marginals *p*(*x*_1_,*y*) and *p*(*x*_2_,*y*), and derives redundancy by subtraction. Because BROJA requires solving a convex optimisation problem over the polytope of consistent distributions [41], it is substantially slower than $I_{\min}$, particularly for variables with many levels. We therefore use $I_{\min}$ for primary analyses and report sensitivity analyses with BROJA and *I*_mmi_ in S1 Appendix. All three measures produced qualitatively similar results. Computations were performed using the dit Python library [42].

We use *I*_syn_ to identify irreducibly joint contributions beyond main effects. Throughout, we report synergy both in absolute bits and as a percentage of TMI, defined as the sum of PID atoms.

#### Assumptions and scope of the PID framework.

PID requires a joint probability mass function *p*(*x*_1_, *x*_2_, *y*) but imposes no parametric assumptions beyond this: it does not presuppose linearity, a particular link function, or a specific noise model. The framework is therefore applicable whenever a discrete (or discretised) joint distribution can be estimated from observed frequencies. Source correlation does not violate any assumption of PID, though it affects which atoms carry information; our simulation study (Section 4.6) confirms accurate recovery under both independent and correlated sources. Continuous PID formulations exist (e.g., Gaussian PID [35,36]), but no mature framework currently handles the mix of binary, ordinal, and continuous variables present in our dataset; see the Study Limitations section for a fuller discussion. Our discretisation strategy handles all variable types within a single estimation framework while producing clinically interpretable categories (Section 4.3.1). Adequate sample sizes are needed to populate joint states of the source–source–target triplet; Section 4.3.1 describes the bias-correction and sparse-state merging procedures we use to address this requirement.

### 4.2 Synergistic information

Synergistic information is the piece of predictive power for a third variable (target) that appears only when two source variables are observed together. If either source is considered in isolation, this component of the signal vanishes. It represents information that is carried jointly by both sources and cannot be recovered from either alone. Consequently, any model that assumes the total effect of two predictors can be written as a sum (additive) or a product (multiplicative/independent-action) will miss that irreducible joint, or synergistic, part.

Mutual information (MI) quantifies how much uncertainty about an outcome is reduced by observing a feature, without assuming a specific model or scale. Partial Information Decomposition (PID) separates the joint information from two features into redundant, unique, and synergistic parts, allowing us to test whether combinations matter beyond their individual contributions.

For example, if PID indicates that 18% of the information predicting an outcome is synergistic, then an additive or multiplicative model cannot recover that 18% from main effects alone; at minimum, an explicit interaction term $\left(X_{1}X_{2}\right)$ or a more flexible model is required to capture the observed pattern.

### 4.3 Screening candidate source–target triplets

Before running PID, we screened every candidate triplet $\left(X_{a},X_{b}\;\to \;Y_{j}\right)$ for evidence of any statistical dependence between the source pair and the target. For each triplet the null hypothesis is


$$\begin{array}{c} H_{0}:I(\{X_{a},X_{b}\};\;Y_{j})=0, \end{array}$$

i.e., the target *Y*_*j*_ is statistically independent of the joint source (*X*_*a*_, *X*_*b*_). The test statistic is the plug-in (maximum-likelihood) estimate of the joint mutual information computed from the observed contingency table. To build a null distribution we performed 500 random permutations of the target labels *Y*_*j*_ while holding the joint distribution of (*X*_*a*_, *X*_*b*_) fixed. Shuffling only the target preserves any dependence structure between the two sources, so the null distribution reflects the absence of genuine source–target information without destroying inter-source associations. For each permutation the plug-in MI was recomputed from the permuted data, and the *p*-value was defined as the proportion of permuted MI values greater than or equal to the observed MI. Across all triplets, we controlled the false-discovery rate at *q* = 0.05 using the Benjamini–Hochberg procedure [43]. Triplets whose adjusted *p*-value fell below 0.05 were retained for subsequent PID estimation; the remainder were excluded, thereby focusing computational resources on triplets with statistically supported source–target dependence.

#### 4.3.1 PID estimation with bias correction.

Plug-in estimates of information-theoretic quantities are positively biased in finite samples. For multivariate measures such as O-information, this bias can distort the balance between redundancy and synergy [44]; for PID atoms specifically, Koçillari et al. [45] showed that the bias of synergy grows *quadratically* with the number of discrete variable levels, whereas biases in unique and redundant information grow more slowly. Correcting for finite-sample bias is therefore especially important for synergy estimation in variables with multiple categories.

The expectation of a plug-in information estimator admits a series expansion in inverse sample size [45]:


$$\begin{array}{c} 𝔼[\hat{I}]\;=\;I_{\infty }\;+\;\frac{a_{1}}{N}\;+\;\frac{a_{2}}{N^{2}}\;+\;O(N^{-3}), \end{array}$$

where $I_{\infty }$ is the population quantity of interest and *a*_1_, *a*_2_ are unknown, distribution-dependent constants. Quadratic extrapolation (QE) exploits this expansion by evaluating the estimator at multiple sample sizes and fitting a degree-2 polynomial in 1/*N*; the intercept recovers $I_{\infty }$, removing both the first- and second-order bias terms. A linear (two-point) extrapolation would leave the $a_{2}/N^{2}$ component uncorrected - a material concern given the quadratic scaling of synergy bias with variable cardinality.

Concretely, for each PID atom we compute the plug-in estimate on three nested samples: the full dataset (*N* observations), a random subsample of *N*/2, and a random subsample of *N*/4. These three evaluation points at 1/*N*, 2/*N* and 4/*N* define a degree-2 polynomial whose intercept (at 1/N→0) is the QE estimate.

To remove any residual estimator-specific bias, we combine QE with a target-shuffle null: we repeat the same three-point QE procedure on data in which the target variable has been randomly permuted (while preserving the joint of the two sources), and subtract the shuffled QE estimate from the original:


$$\begin{array}{c} {PID}_{QE+SH}=QE\;\left({PID}_{orig}\right)-QE\;\left({PID}_{shuffled}\right). \end{array}$$

By shuffling only the target while keeping the sources fixed, the joint dependency structure among sources is preserved. This ensures that the null distribution reflects the absence of genuine source–target information without destroying the joint information shared between sources. We average over *K* = 30 independent target shuffles per subsample fraction to stabilise the null estimate.

We truncate negative atoms to zero for reporting. This preserves the non-negativity of reported synergies but breaks exact additivity of PID atoms, and it can inflate the prevalence of very small positive synergies near zero.

### 4.4 Breadth–Uniformity–Synergy–Total (BUST) classification

For each unordered source pair (*X*_*a*_, *X*_*b*_) we ask two related questions: (i) is its *synergy* spread across many targets (broad) or concentrated on a few (narrow)? (ii) are those synergistic effects similar across population strata (uniform) or do they vary (specific)? We answer (i) by quantifying how the pair’s synergy is *distributed* across targets and (ii) by quantifying how *even* that distribution is across strata. Both quantities are defined in terms of normalised synergy-based probability distributions and summarised using bounded deviation-from-uniformity measures.

#### Notation.

Let J denote the set of all targets. For each unordered pair (*X*_*a*_, *X*_*b*_) and target j∈J, let *s*_*j*_ ≥ 0 be the bias-corrected PID synergy (Section 4.3.1), with $s_{j}=0$ when no positive synergy is detected. The pair’s total synergy strength is


$$\begin{array}{c} S\;=\;\sum_{j\in J}s_{j}, \end{array}$$

and the associated *synergy shares* across targets are


$$\begin{array}{c} w_{j}\;=\;\frac{s_{j}}{S},\;j\in J,\;\sum_{j}w_{j}=1, \end{array}$$

defined whenever *S* > 0. For stratified analyses, let $s_{j}^{\left(s\right)}\geq 0$ and $\overset{\left(s\right)}{\underset{j}{TMI}}\geq 0$ denote the synergy and TMI (sum of all PID atoms) for target *j* in stratum *s* of stratification type *t* (sex, multimorbidity, or age group).

#### Breadth (B): synergy spread across targets.

We quantify how broadly synergy is distributed over targets using the normalised Shannon entropy of the synergy shares $\{w_{j}{\}}_{j\in J}$. For each pair with *S* > 0 we define


$$\begin{array}{c} H_{B}\;=\;-\frac{\sum_{j\in J}w_{j}\log w_{j}}{\log|J|}\;\in \;[0,1], \end{array}$$

with $H_{B}=0$ when all synergy is concentrated on a single target and $H_{B}=1$ when synergy is perfectly uniform across all targets. To obtain a median-centred score that reflects the empirical behaviour of the data, we compute the median breadth across all pairs, *H*_*B*,med_, and map *H*_*B*_ to a breadth score


$$\begin{array}{c} B\;=\;2\;\left(\frac{H_{B}}{H_{B,med}}-1\right), \end{array}$$

clipped to [−1, 1] for stability. Thus B≈+1 denotes broadly distributed synergy (generalist behaviour), B≈−1 denotes highly concentrated synergy (specialist behaviour), and B≈0 corresponds to the median breadth observed in the data. Pairs with *S*=0 do not receive a B score and are excluded from subsequent BUST plots.

As robustness checks, we also compute alternative breadth measures by replacing *H*_*B*_ with (i) 1 minus a normalised Gini coefficient of the synergy shares, and (ii) 1 minus a normalised Kolmogorov–Smirnov (KS) distance between the empirical cumulative distribution of *w*_*j*_ and the uniform distribution. These alternative B scores are reported in S1 Appendix and are highly correlated with the entropy-based B.

#### Uniformity (U): cross-stratum stability of synergy.

To characterise whether a pair’s synergy is uniform across strata or context-specific, we first form synergy-based distributions across strata for each target and stratification type. For target *j* and stratum *s* of type *t* we define the *synergy fraction*


$$\begin{array}{c} \pi_{j}^{\left(s,t\right)}\;=\;\left\{ \begin{array}{cc} s_{j}^{\left(s\right)}/\overset{\left(s\right)}{\underset{j}{TMI}}, & \overset{\left(s\right)}{\underset{j}{TMI}}>0,\\ {}[4pt]0, & \overset{\left(s\right)}{\underset{j}{TMI}}=0, \end{array}\right. \end{array}$$

so that $\pi_{j}^{\left(s,t\right)}\in [0,1]$ whenever defined. For a given *j* and *t*, we collect the fractions $\left\{\pi_{j}^{\left(s,t\right)}:s\in S_{t}\right\}$ across s*t*rata, add a small stabilising constant ε to each value to avoid zero cells, and normalise to obtain a probability vector


$$\begin{array}{c} u_{j}^{\left(s,t\right)}\;=\;\frac{\pi_{j}^{\left(s,t\right)}+\varepsilon }{\sum_{r\in S_{t}}(\pi_{j}^{\left(r,t\right)}+\varepsilon )},\;s\in S_{t}, \end{array}$$

whenever at least two strata contribute non-zero information. For each target–type combination we then compute the normalised entropy


$$\begin{array}{c} H_{U,j}^{\left(t\right)}\;=\;-\frac{\sum_{s\in S_{t}}u_{j}^{\left(s,t\right)}\log u_{j}^{\left(s,t\right)}}{\log|S_{t}|}\;\in \;[0,1], \end{array}$$

so that $H_{U,j}^{\left(t\right)}\approx 1$ when synergy fractions are similar across strata of type *t* (uniform), and $H_{U,j}^{\left(t\right)}\approx 0$ when synergy is concentrated in a subset of s*t*rata (specific to, e.g., a particular age group or sex).

Because some targets contribute little synergy overall and may be noisy, we aggregate these target-level uniformities using weights proportional to their full-population synergy *s*_*j*_. For each stratification type *t* we define


$$\begin{array}{c} H_{U}^{\left(t\right)}\;=\;\frac{\sum_{j}s_{j}\;H_{U,j}^{\left(t\right)}}{\sum_{j}s_{j}}, \end{array}$$

summing over targets with well-defined $H_{U,j}^{\left(t\right)}$ and *s*_*j*_ > 0. The overall uniformity index for a pair is then obtained by averaging across available stratification types:


$$\begin{array}{c} H_{U}\;=\;\frac{1}{\left|T^{*}\right|}\sum_{t\in T^{*}}H_{U}^{\left(t\right)}, \end{array}$$

where $T^{*}$ is the subset of types (sex, multimorbidity, age group) with at least two informative strata. As for breadth, we median-centre this raw uniformity index using its empirical median *H*_*U*,med_ across pairs and define


$$\begin{array}{c} U\;=\;2\;\left(\frac{H_{U}}{H_{U,med}}-1\right), \end{array}$$

clipped to [−1, 1]. Large positive *U* values indicate synergy fractions that are stable across strata (uniform, or “universal”), whereas large negative *U* values indicate synergy that is concentrated in specific demographic or strata (context-specific).

To directly address potential numerical instabilities of coefficient-of-variation based metrics when means are small, all *U* scores are built from bounded probability distributions $\left\{u_{j}^{\left(s,t\right)}\right\}$ with explicit ε-stabilisation, avoiding divisions by near-zero denominators. In sensitivity analyses we recompute uniformity using (i) 1 minus a normalised Gini coefficient and (ii) 1 minus a normalised KS distance for the $\left\{u_{j}^{\left(s,t\right)}\right\}$, rather than entropy. These alternative U measures show high correlations with the entropy-based U and do not materially change the BUST map.

#### Synergy strength (S) and total information (T).

For each pair, the *synergy strength* is defined as the total amount of positive synergy across all targets,


$$\begin{array}{c} S\;=\;\sum_{j\in J}s_{j}, \end{array}$$

measured in bits. The *total information* (T) is defined by summing TMI_*j*_ over all targets with non-zero synergy in the full population,


$$\begin{array}{c} T\;=\;\sum_{j\in J}{TMI}_{j}, \end{array}$$

thereby capturing the overall information content carried by the sources about the outcome space, irrespective of whether it is synergistic, redundant, or unique. The BUST map uses (*B*,*U*) as a 2D embedding and encodes *S* (and, where available, *T*) through bubble size and colour to visualise which pairs are both broad, uniform and information-rich.

### 4.5 Analysing BUST networks

#### Composite score construction.

To prioritise source pairs that are simultaneously broad, cross-stratum robust, strongly synergistic, and information-rich, we constructed a composite BUST score for network analysis. First, the breadth and uniformity scores (*B*,*U*) obtained from the entropy-based metrics were already scaled to [−1, 1] as described above. Second, synergy strength *S* and total information *T* were each transformed to [−1, 1] by median-centring and applying a hyperbolic tangent transform to soften the influence of extreme values:


$$\begin{array}{c} S^{*}\;=\;\tanh\;\left(2\left(\frac{S}{median(S)}-1\right)\right),\;T^{*}\;=\;\tanh\;\left(2\left(\frac{T}{median(T)}-1\right)\right), \end{array}$$

with $T^{*}$ omitted if reliable TMI was unavailable. All four dimensions were then linearly mapped from [−1, 1] to [0,1] and combined multiplicatively,


$$\begin{array}{c} {BUST}_{comp}\;=\;B_{01}\times U_{01}\times S_{01}\times T_{01}, \end{array}$$

where each subscript 01 denotes the corresponding rescaled value in [0,1]. The multiplicative form acts as a soft *AND-gate*: a pair must score reasonably well on *all* dimensions (breadth, uniformity, synergy strength and total information) to attain a high composite value, while a low score in any dimension down-weights the overall importance. This avoids selecting pairs that are extreme on a single axis but weak on the others, and reflects our interest in identifying edges that are jointly broad, robust and information-rich. For network visualisations we defined “strong” BUST edges as those with composite scores above the 75th percentile of BUST_comp_, and we confirmed that the resulting network structure is robust to alternative percentile cut-offs and to using Gini- or KS-based B and U in place of the entropy-based metrics (Section S2 of S1 Appendix).

#### Synergistic clique detection and stability analysis.

Stable synergistic cliques were identified using a threshold-based stability approach to avoid arbitrary cutoffs. We constructed a weighted graph where nodes represented variables and edges were weighted by the composite BUST scores integrating breadth, uniformity, synergy strength and total information (Section 4.5). Cliques (fully connected subgraphs of size ≥3) were enumerated independently at four thresholds (67th, 75th, 80th, 85th percentiles of composite scores). Cliques appearing in ≥3/4 thresholds were classified as stable. Each stable clique was characterised by its mean composite weight, average B and U scores, and total synergy strength, then ranked by a weighted importance score


$$\begin{array}{c} Imp\;=\;0.4\times stability\;+\;0.3\times size\;+\;0.3\times mean\;weight. \end{array}$$

This choice reflects three design considerations. First, we prioritised *stability* across thresholds (weight 0.4), because cliques that recur under different cut-offs are less likely to be artefacts of a particular threshold. Second, we favoured cliques that are *larger* (weight 0.3), as these capture broader constellations of jointly interacting variables. Third, we gave comparable weight to the *mean composite edge weight* (weight 0.3), so that cliques with consistently strong BUST edges are preferred over those formed by weaker links. These weights are heuristic but reflect a conservative preference for robust, sizeable and strongly connected cliques rather than small or unstable motifs driven by a single strong edge.

#### Rule-based characterisation of joint-state behaviour.

To characterise how BUST-identified synergistic constellations behaved with respect to individual outcomes, we examined the distribution of each target variable across all observed joint states of the constellation variables. For each constellation–target combination, we fitted an ordinal regression model with the target as the response and the constellation variables as main effects, and used the model to compute the expected value of the target under an additive assumption. We then quantified, for each joint state, the deviation between its observed mean and this additive prediction. Joint states whose absolute deviation lay in the upper 5% were labelled rule-like configurations. To assess whether these configurations contributed information beyond the main effects, we refitted the ordinal models with binary indicators for the rule-like states and compared model fits using the Akaike Information Criterion. Detailed results for each constellation and target are provided in S1 Appendix.

### 4.6 Simulation validation of the estimation pipeline

To verify that the QE bias-corrected PID estimation pipeline (Section 4.3.1) and the BUST summary scores (Section 4.4) recover known ground truths under realistic conditions, we conducted three sets of simulation experiments with analytically defined joint distributions whose PID atoms were computed numerically using the dit library [42].

#### Cardinality sweep.

We varied the number of levels per variable (K∈{2,3,4,5}) across four deterministic target functions (modular copy, XOR, minimum, and a redundancy-dominated function) at two source-coupling strengths (*c* = 0 and *c* = 0.5) and four sample sizes (N∈{500,1,000,2,000,2,636}, where *N* = 2,636 corresponds to one of the age-group strata in the LASA analyses). For each of the 128 conditions, 50 independent replicates were drawn.

#### LASA-matched cardinality profiles.

To assess performance under the asymmetric cardinalities present in the empirical data, we constructed four profiles matching common LASA variable-cardinality combinations (3×4→5, 2×5→5, 3×7→5, 5×7→5) under three information regimes (synergy-rich, function-of-one, and mixed).

#### BUST score recovery.

We further tested whether the breadth (*B*), synergy strength (*S*), and total information (*T*) BUST summary scores were faithfully recovered from estimated (rather than ground-truth) PID atoms, using three synthetic source pairs with known breadth profiles across eight targets.

#### Bootstrap confidence intervals.

To quantify sampling variability, we computed percentile bootstrap 95% confidence intervals for the top-10 BUST-ranked source pairs (1,000 replicates, resampling the full-population dataset with replacement and re-estimating QE-corrected PID atoms per replicate). Full details and results are provided in Section S7 of S1 Appendix.

Results of the simulation experiments are presented in Section 2.2; full figures are in Section S5 of S1 Appendix.

### 4.7 Sensitivity to discretisation choices

To assess whether the BUST classification is sensitive to the choice of discretisation strategy, we re-ran the full PID pipeline on the LASA full-population dataset under five alternative binning schemes: (i) the clinical cut-points used in the primary analysis; (ii) equal-frequency tertiles; (iii) equal-frequency quartiles; (iv) equal-width terciles; and (v) a coarsened binary split at each variable’s median. For each scheme, we recomputed all PID atoms with QE bias correction, constructed BUST scores, and compared the resulting pair rankings using Spearman rank correlations of composite BUST scores and Jaccard overlap of the top-10 ranked pairs. Among the four non-binary schemes, Spearman correlations ranged from 0.83 to 0.96, and the equal-frequency tertile and quartile schemes were nearly interchangeable (ρ=0.96, Jaccard top-10 = 0.82). The coarsened binary scheme was poorly correlated with all others (ρ=0.06−−0.18), consistent with the severe information loss from collapsing multi-level variables to two categories. Full results, including BUST map comparisons and rank correlation matrices, are provided in Section S6 of S1 Appendix.

### 4.8 Data and preprocessing

We analysed data from the Longitudinal Ageing Study Amsterdam (LASA), an ongoing population-based cohort designed to study physical, emotional, cognitive and social functioning in older adults in the Netherlands. The original cohort of 3,107 community-dwelling adults aged 55–85 years was recruited in 1992/93 and has been followed triennially ever since, with refreshment samples drawn in 2002/03 (n = 1,002) and 2012/13 (n = 1,023) to maintain population representativeness [17]. Data are collected through face-to-face structured interviews and self-completed questionnaires.

For the present analyses, we used data from the 1995/96–2021/22 waves (nine waves, each approximately three years apart). After excluding interviews with more than 50% missing values and applying random-forest imputation (see below), the analytic sample comprised 14,716 person-wave observations from 4,025 unique participants. We focused on variables that are central to multimorbidity and ageing research and that appear throughout the results: symptoms (sleep problems, pain), clinical signs (grip strength, body mass index, waist–hip ratio, mean arterial pressure), behaviours (physical activity, alcohol use), self-rated health indicators (perceived general health, perceived health relative to peers), cognition (MMSE score, memory complaints, memory problems), sensory function (hearing difficulties) and activities of daily living (functional limitations), anxiety and depression.

#### Functional limitations.

Functional limitations were assessed with six activities of daily living (ADL): climbing a flight of stairs, dressing/undressing, rising from a chair, cutting toenails, walking outdoors for five minutes, and using transportation. Each item was scored on a 5-point ordinal scale (0 = “yes, without difficulty” to 4 = “no, cannot”). We computed a total score by summing the six items (range 0–24; higher = worse function). To improve interpretability while maintaining gradation, we defined a five-level variable: “none” (total = 0) and quartiles of positive distribution among participants with any limitation (total > 0).

#### Discretisation of symptoms and signs.

All variables were analysed as discrete states. Where available, we discretised continuous measures using clinically interpretable cut-points; otherwise we retained the native ordinal response categories as described in LASA documentation. To ensure adequate support for information-theoretic estimation in three-way contingency tables, we applied an additional deterministic post-processing step that merges adjacent categories to eliminate sparse observed triplet states to ensure no observed triplet state had fewer than 5 participants.

Body mass index (BMI) was categorised following the World Health Organization classifications [46]: under/normal weight (<24.9 *kg*/*m*^2^), overweight $\left(25.0-29.9kg/m^{2}\right)$, and obesity $\left(\geq 30.0kg/m^{2}\right)$. The sleep quality measure, derived by summing answers to three categorical questions (problems falling asleep, waking through the night and waking up too early), ranged from 3 (no problems) to 12 (many problems). To reduce the sparsity in the data, the lowest two scores were combined and the upper tail collapsed giving states: 3–4, 5, 6, 7, 8, 9, and ≥10 (i.e., 10–12 combined). For dominant grip strength, we implemented a two-step process based on the EWGSOP2 sarcopenia guidelines: individuals with grip strength below 27 kg for men and below 16 kg for women were classified as having ‘Low’ strength, and those with values at or above these thresholds were subsequently divided into quartiles by sex to capture further gradations in strength [47], with the top two quartiles combined due to data sparsity. Cognition was assessed with the Mini-Mental State Examination (MMSE; 0–30). Following LASA documentation, we computed the total by summing 22 items and using the higher of the two attention/calculation alternatives (serial 7s or spelling ‘WORLD’ backwards), assigning 0 to missing items and withholding a total if >6 items were missing. We then categorised cognition into strata: impaired (0–23) versus no impairment (24–30). Pain was measured with the LASA 6-item Nottingham Health Profile subscale (standing, changing position, sitting, walking, constant pain; the “unbearable pain” item is excluded from scoring). Responses were dichotomous (yes/no) [48]. Following LASA documentation, we recoded no=1 and yes=2, summed the five retained items with single-item mean imputation when one response was missing (yielding a 5-10 score), and then linearly rescaled. The the two highest levels were combined, yielding five ordered levels (1, 2, 3, 4, and ≥5), where higher scores mean more pain symptoms. Mean arterial pressure (MAP; blood pressure) was categorised as normal (<93 mmHg) versus elevated/hypertensive (≥93 mmHg). Physical activity was assessed using a standard LASA activity scale and discretised into tertiles. Alcohol use was categorised into abstinent, light/moderate and heavy use based on weekly consumption. Perception of general health was retained as an ordinal measure, with the two highest response categories combined (giving three ordered levels). Similarly, perception of health compared to peers were both retained as ordinal measures, with the two lowest response categories combined to give three ordered levels. Depressive symptoms was assessed with the Center for Epidemiologic Studies Depression Scale (CES D), grouped into two ordered categories: no/mild symptoms (0–19) and moderate/severe (≥20) [49]. Anxiety symptoms were identified using the anxiety subscale of the Hospital Anxiety and Depression Scale (HADS), grouped two ordered categories: minimal (0–3) and elevated (≥4) [50]. Memory complaints were determined by a yes/no response to the question: “Do you have complaints about your memory?”. Similarly, Memory problems were determined by a yes/no response to the question: “Did you consult a doctor about your memory problems?”.

#### Missing data.

Missing data were imputed prior to discretisation. Known invalid responses were treated as missing, and interviews with more than 50% missing values across relevant variables were excluded to minimise any imputation bias. Random forest–based imputation was used because it can accommodate complex nonlinear relations. Continuous and categorical variables were imputed using separate models (regressor vs classifier), each with a maximum tree depth of 5, a minimum of 10 samples per leaf and 100 trees.

#### Post-hoc merging of sparse categories.

After applying the clinically motivated discretisation cut-points described above, we performed an additional merging step to reduce sparsity in higher-order joint states required for information-theoretic estimation. Using the full-sample dataset, we enumerated all variable triplets and computed the observed 3-way joint frequency table for each triplet (i.e., counts of each observed joint category combination within the triplet). We required every observed 3-way joint state to have at least 5 observations. If any triplet contained joint states with count ≤5, we iteratively selected the single *variable–state* pair that appeared in the largest number of sparse triplets and merged that state with an adjacent ordinal neighbour (lower or higher category). When both neighbours were available, the merge direction was chosen to preferentially combine with the neighbour that was itself involved in more sparse triplets. This procedure was repeated until no observed 3-way joint state had fewer than five samples.

#### Stratification.

All analyses were repeated in strata defined *a priori* by age, sex and multimorbidity to assess whether patterns of synergy were uniform or population-specific. Multimorbidity was encoded as a binary variable, with multimorbidity marked present when a person reported two or more chronic diseases.

#### Variables included in the synergy analysis.

All variables except age, sex and multimorbidity (used solely for stratification) were included as candidates for both predictors and outcomes in the PID triplets. Treating variables symmetrically allows estimation of how any pair jointly informs each remaining variable, supporting the Breadth, Uniformity and Total components of the BUST representation. Table 1 summarises all variables included in the synergy analysis together with their domain classification, number of discrete levels after post-hoc merging (Section 4.3.1), and discretisation basis.

**Table 1:** Clinical variables included in the PID synergy analysis.

Variable	Domain	Levels	Discretisation basis
Sleep quality	Symptom	7	Sum of 3 items; tail-collapsed
Pain	Symptom	5	NHP subscale; rescaled sum
Anxiety	Symptom	2	HADS anxiety subscale
Depression	Symptom	2	CES-D threshold
Hearing difficulties	Sensory	3	Self-report ordinal
Memory complaints	Cognition	2	Self-report (yes/no)
Memory problems	Cognition	2	Self-report (yes/no)
MMSE (cognition)	Cognition	2	MMSE total; clinical cutpoint
Grip strength	Sign	4	EWGSOP2 cutpoints + sex-specific quartiles
BMI	Sign	3	WHO classification
WHR obesity	Sign	2	Clinical cutpoint
Blood pressure (MAP)	Sign	2	Normal vs. elevated/hypertensive
Physical activity	Behaviour	3	LASA activity scale; tertiles
Alcohol use	Behaviour	3	Weekly consumption categories
Perceived general health	Self-rated health	3	Ordinal; top categories combined
Health vs. peers	Self-rated health	3	Ordinal; bottom categories combined
Functional limitations	ADL	3	ADL sum score; none vs. quartiles

*Levels* refers to the number of discrete categories after post-hoc sparse-state merging.

Abbreviations: ADL = activities of daily living; BMI = body mass index; CES-D = Center for Epidemiologic Studies Depression Scale; EWGSOP2 = European Working Group on Sarcopenia in Older People 2; HADS = Hospital Anxiety and Depression Scale; MAP = mean arterial pressure; MMSE = Mini-Mental State Examination; NHP = Nottingham Health Profile; WHO = World Health Organization; WHR = waist–hip ratio.

Memory complaints, memory problems, and MMSE are grouped under “Cognition” for analytic parsimony. Alternative classifications are defensible (memory complaints as a symptom, MMSE as a clinical sign, doctor-confirmed memory problems as a disease); the choice does not affect downstream PID or BUST calculations.

### Data and code availability

The LASA data used in this study are not publicly available because they contain sensitive participant information and are distributed under governed access. Researchers may request access to LASA data for specific research questions by submitting an analysis proposal to the LASA Steering Group. If approved, access is provided under a data-sharing agreement. Details and the required proposal materials are available via the LASA website: https://lasa-vu.nl/en/request-data/.

The analysis pipeline is publicly available as the open-source Python package bust-pid at https://github.com/cillianhourican/bust-pid. The package implements permutation screening, QE-corrected PID estimation, BUST scoring, network construction, and visualisation, and includes reproduction scripts for the main paper figures (requiring LASA data access) as well as a synthetic-data demo.

## Supporting information

S1 Appendix Supplementary methods, tables and figures. Contains: (S1) number of source–source–target triplets with significant joint mutual information by stratum; (S2) robustness of the breadth and uniformity metrics to alternative definitions; (S3) prediction surfaces comparing observed, additive, and multiplicative baselines for representative triplets; (S4) rule-based joint-state patterns; (S5) simulation validation of the QE-bias-corrected PID estimation pipeline, including cardinality sweeps, LASA-matched cardinality profiles, and BUST-score recovery; (S6) sensitivity of the BUST classification to discretisation choices; and (S7) bootstrap 95% confidence intervals for synergy estimates of the top-10 BUST pairs.
(PDF)

S1 File Reproducibility package. *PID_BUST_Results_and_DemoScripts.zip* is a self-contained archive enabling readers to reproduce the main-text analyses and figures from pre-computed PID results. It contains a Jupyter notebook that regenerates Figs 1–4, plotting utility modules, pre-computed permutation-screening results and analysis-ready dataframes, BUST-network artefacts (edge lists, node attributes, clique catalogues, and quadrant assignments), hub-variable summaries, and a Python requirements file. Installation and execution are documented in the accompanying README. Raw LASA participant data are not included (governed access); the package provides the full downstream pipeline conditional on data access.
(ZIP)
