# Supplementary material for: Interpreting higher-order dependence in multimorbidity using cohort data: A partial information decomposition approach
Source: PLoS Comput Biol. 2026 Jun 10;22(6):e1014386. doi: 10.1371/journal.pcbi.1014386 (PMC13268148; doi:10.1371/journal.pcbi.1014386)
Supplement: S1 Appendix — Contains: (S1) number of source–source–target triplets with significant joint mutual information by stratum; (S2) robustness of the breadth and uniformity metrics to alternative definitions; (S3) prediction surfaces comparing observed, additive, and multiplicative baselines for representative triplets; (S4) rule-based joint-state patterns; (S5) simulation validation of the QE-bias-corrected PID estimation pipeline, including cardinality sweeps, LASA-matched cardinality profiles, and BUST-score recovery; (S6) sensitivity of the BUST classification to discretisation choices; and (S7) bootstrap 95% confidence intervals for synergy estimates of the top-10 BUST pairs. (PDF) [file pcbi.1014386.s001.pdf]

S1 Appendix

Supporting Information for
Interpreting Higher-Order Dependence in Multimorbidity using Cohort Data:
A Partial Information Decomposition Approach

Supplementary Materials

S1. Joint Mutual Information Significance in LASA

Table A: Number of source–source–target triplets with significant joint mutual information (FDR $q = 0.05$) by stratum.

Dataset	Significant / total	Percent significant	FDR critical p
AgeGroup_0	1799/2040	88.2%	0.0439
AgeGroup_1	1901/2040	93.2%	0.0459
AgeGroup_2	1957/2040	95.9%	0.0479
AgeGroup_3	1750/2040	85.8%	0.0419
Multimorbidity_0	1930/2040	94.6%	0.0439
Multimorbidity_1	1917/2040	93.9%	0.0439
Sex_0	1978/2040	97.0%	0.0479
Sex_1	1901/2040	93.2%	0.0459
Full population	2005/2040	98.3%	0.0459

S2. Robustness of breadth and uniformity metrics

The main BUST map (see the BUST summary scores section of the main paper) uses entropy-based measures of breadth and uniformity: normalised Shannon entropy of synergy shares across targets for breadth (H_B), and normalised entropy of synergy fractions across strata for uniformity (H_U). To assess whether our conclusions depend on this particular deviation-from-uniformity metric, we repeated all calculations using two alternative families of indices applied to the *same* synergy-based probability distributions: (i) a Gini-based measure, and (ii) a Kolmogorov–Smirnov (KS)-based measure.

Gini-based indices. Let $\mathbf{p} = (p_1, \ldots, p_n)$ denote a non-negative weight vector with $\sum_i p_i = 1$, representing either synergy shares across targets or stabilised synergy fractions across strata (as in the main paper's BUST summary scores section). Let $p_{(1)} \leq \cdots \leq p_{(n)}$ denote the components sorted in ascending order. We compute the standard Gini coefficient

$$G(\mathbf{p}) = \frac{2}{n} \frac{\sum_{i=1}^{n} i \, p_{(i)}}{\sum_{i=1}^{n} p_{(i)}} - \frac{n+1}{n},$$

which lies in $[0,(n-1)/n]$ for a probability vector. To place breadth and uniformity on a $[0,1]$ scale where larger values indicate greater evenness (rather than inequality), we first normalise by the maximal value $G_{\max} = (n-1)/n$ and define

$$G_{\text{norm}}(\mathbf{p}) \;=\; \frac{G(\mathbf{p})}{G_{\max}}, \qquad \tilde{E}_{\text{Gini}}(\mathbf{p}) \;=\; 1 - G_{\text{norm}}(\mathbf{p}) \in [0,1].$$

We then substitute $\tilde{E}_{\text{Gini}}$ in place of H_B or H_U and apply the same median-centring to $[-1,1]$ as in the main text, yielding Gini-based breadth and uniformity scores B_{Gini} and U_{Gini}.

KS-based indices. As a second alternative, we quantified deviation from uniformity using a discrete KS distance. For a probability vector $\mathbf{p}$ (as above), we sort components in *descending* order, $p_{[1]} \geq \cdots \geq p_{[n]}$, and define their cumulative sums $F_{\mathbf{p}}(k) = \sum_{i=1}^{k} p_{[i]}$ for $k = 1,\ldots,n$. The corresponding uniform cumulative distribution is $F_{\text{unif}}(k) = k/n$. The (one-sample) KS distance from uniformity is

$$D(\mathbf{p}) \;=\; \max_{1\leq k\leq n} \left| F_{\mathbf{p}}(k) - \frac{k}{n} \right|,$$

which attains its maximum possible value $D_{\max} = 1 - 1/n$ for a probability vector concentrated entirely on a single category. We normalise and invert this to obtain

$$D_{\text{norm}}(\mathbf{p}) \;=\; \frac{D(\mathbf{p})}{D_{\max}}, \qquad \tilde{E}_{\text{KS}}(\mathbf{p}) \;=\; 1 - D_{\text{norm}}(\mathbf{p}) \in [0,1],$$

and again substitute $\tilde{E}_{\text{KS}}$ for H_B or H_U, followed by the same median-centring to generate KS-based breadth and uniformity scores B_{KS} and U_{KS}.

Empirical agreement of entropy-, Gini-, and KS-based metrics. We computed entropy-, Gini-, and KS-based breadth and uniformity metrics for all source pairs with non-zero synergy in the full population ($N = 135$ pairs with complete data). The three breadth indices were highly correlated: pairwise Pearson correlations between H_B, $\tilde{E}_{\text{Gini}}$, and $\tilde{E}_{\text{KS}}$ ranged from $r = 0.93$ to $r = 0.97$, and Spearman rank correlations from $\rho = 0.95$ to $\rho = 0.99$. Uniformity indices showed even stronger agreement: pairwise Pearson correlations between H_U, $\tilde{E}_{\text{Gini}}$, and $\tilde{E}_{\text{KS}}$ were all ≥ 0.98, with Spearman correlations ≥ 0.97. Thus, entropy-, Gini-, and KS-based measures yield almost identical rankings of source pairs by breadth and by uniformity.

Because all three families of indices operate on the same synergy-based probability distributions and exhibit near-perfect agreement, our substantive conclusions about which pairs are broad vs. narrow and uniform vs. specific, and about the overall structure of the BUST map and network, are robust to the particular choice of deviation-from-uniformity measure.

Concordance across redundancy definitions. To verify that our findings are not driven by the choice of redundancy measure, we repeated the full PID estimation for all source–target triplets in the full population using three definitions: I_{min} (Williams–Beer; primary), I_{mmi} (minimum mutual information), and BROJA (Bertschinger et al.). Spearman rank correlations of total synergy strength across all 136 source pairs were $\rho = 0.998$ (I_{min} vs. I_{mmi}), $\rho = 0.989$ (I_{min} vs. BROJA), and $\rho = 0.986$ (I_{mmi} vs. BROJA; all $p < 10^{-10}$). The four highest-ranked pairs by total synergy - Pain–Physical Activity, Alcohol Use–Grip Strength, Health vs. Peers–Physical Activity, and Health vs. Peers–Functional Limitations - appeared in the top five under all three measures. BROJA synergy estimates were systematically lower in magnitude (consistent with its tighter redundancy definition) but preserved the same relative ordering. These results confirm that the qualitative conclusions and pair rankings reported in the main text are robust to the choice of redundancy definition.

S3. Prediction surfaces and interpretation

Comparing synergy with additive and multiplicative baselines

Additive and multiplicative models encode built-in constraints: additive models force the joint effect to be a sum of main effects; multiplicative or independent-action models become additive on a log scale and likewise contain no irreducible joint term. When the only informative component is jointly available from two features, both families show a systematic lack-of-fit even if the main effects are strong. Table B summarises these constraints and what to expect in the residuals when there is synergy.

We can create residual maps to compare triplets with and without synergy. For each triplet $(X, Z \to Y)$ we form a grid from the discrete levels of X and Z to get every possible combination of variables in their joint state space. Let i index levels of X and j index levels of Z. Define $\mu_{ij} = \mathbb{E}[Y \mid X = i, Z = j]$ as the observed mean outcome for the combination $X = i$ and $Z = j$. Let $\bar{Y}_{i\cdot}$, $\bar{Y}_{\cdot j}$, and $\bar{Y}_{\cdot\cdot}$ be the count-weighted row, column, and grand means. The *additive* expectation and residual are

$$\widehat{\mu}_{ij}^{\text{add}} = \bar{Y}_{i\cdot} + \bar{Y}_{\cdot j} - \bar{Y}_{\cdot\cdot}, \qquad R_{ij}^{\text{add}} = \mu_{ij} - \widehat{\mu}_{ij}^{\text{add}}.$$

Here $R_{ij}^{\text{add}} > 0$ indicates outcomes worse than the additive expectation for that $X = i$, $Z = j$ combination; $R_{ij}^{\text{add}} < 0$ indicates better than additive.

To assess a *multiplicative* (independent-action) baseline, we operate on the log scale and add a small ε to avoid zeros:

$$\widehat{\log \mu_{ij}}^{\text{mult}} = \log \bar{Y}_{i\cdot} + \log \bar{Y}_{\cdot j} - \log \bar{Y}_{\cdot\cdot}, \qquad L_{ij}^{\text{mult}} = \log \mu_{ij} - \widehat{\log \mu_{ij}}^{\text{mult}},$$

so that $\exp\big(L_{ij}^{\text{mult}}\big)$ is the observed/expected ratio under multiplicativity (values > 1 denote worse-than-multiplicative outcomes).

For a set of representative triplets, we display heatmaps of the observed means μ_{ij}, the additive expectations $\widehat{\mu}_{ij}^{\text{add}}$, and the additive residuals R_{ij}^{add} to show deviations from model expectations. Multiplicative log-residual maps L_{ij}^{mult} are provided in the Supplement and are interpreted analogously.

Table B: Synergy and built-in constraints in two common model families

**Model family**	**Built-in mathematical constraint**	**What happens if synergy exists?**
Additive (e.g., linear regression, main-effects GLM)	Effect $= \beta_0 + \beta_1 X_1 + \beta_2 X_2$	Interaction term is forced to 0; the model either pushes synergy into the error term or distorts the β-coefficients.
Multiplicative / independent-action (e.g., log-transformed models)	Effect $= \alpha\, X_1 X_2 \longrightarrow$ additive on the log scale	Still no irreducible joint term; systematic lack-of-fit appears in residual plots or curvature.

Synergistic example (Sleep × WHR → Function): multiplicative view

Fig A shows a representative synergistic triplet (Sleep × WHR → Function) under a multiplicative (independent-action) baseline on the log scale. The log-residual map $L_{ij}^{\text{mult}} = \log \mu_{ij} - \widehat{\log \mu_{ij}}^{\text{mult}}$ highlights a local pocket of *positive* residuals at poor sleep and moderate WHR; $\exp(L_{ij}^{\text{mult}}) > 1$ indicates that observed functional limitation exceeds multiplicative expectations. This pattern mirrors the additive residual pocket in the main figure and is consistent with strictly positive PID synergy for Sleep-WHR with respect to Function. In short, both additive and multiplicative baselines leave structured errors that are explained by an irreducibly joint component.

Non-synergistic example (blood pressure (MAP) × Hearing → Function): additive and multiplicative views

Figs B and C display, respectively, the additive residuals R_{ij}^{add} and the multiplicative log-residuals L_{ij}^{mult} for the blood pressure-Hearing pair. Both maps are near-symmetric around zero with low amplitude and no coherent spatial structure. PID synergy for this triplet is negligible, in agreement with the flat residual surfaces. Here, main-effects models adequately capture the association with Function; adding an interaction would be unlikely to improve fit meaningfully.

Practical reading of residual maps

For additive residuals, $R_{ij}^{\text{add}} > 0$ indicates worse-than-additive outcomes (joint effect larger than the sum of parts), whereas $R_{ij}^{\text{add}} < 0$ indicates better-than-additive outcomes. For multiplicative residuals, values $\exp(L_{ij}^{\text{mult}}) > 1$ (or $L_{ij}^{\text{mult}} > 0$) indicate observed/expected ratios above one under independence, and < 1 indicates the opposite. Residual maps should be interpreted jointly with PID results: structured, localized departures in either residual surface typically coincide with positive synergy, while uniformly pale maps coincide with near-zero synergy. As discussed in Methods, colour scales are centred at zero and cell means are weighted by counts to avoid artifacts from sparse bins.

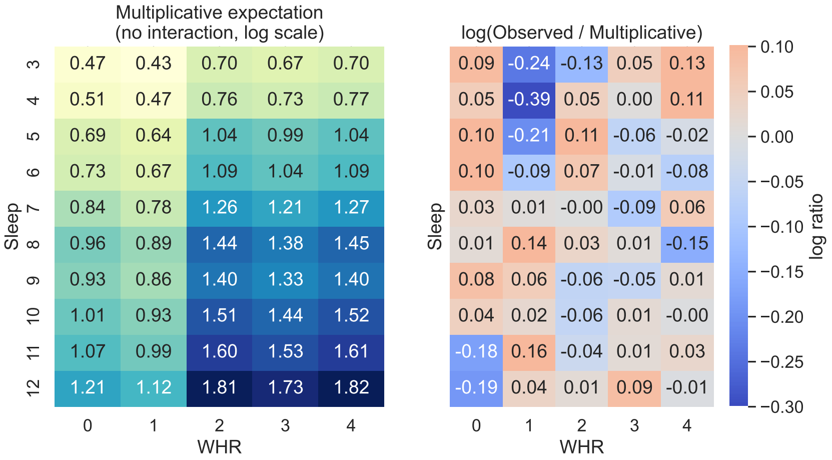

Sleep \ WHR	0	1	2	3	4
3	0.47	0.43	0.70	0.67	0.70
4	0.51	0.47	0.76	0.73	0.77
5	0.69	0.64	1.04	0.99	1.04
6	0.73	0.67	1.09	1.04	1.09
7	0.84	0.78	1.26	1.21	1.27
8	0.96	0.89	1.44	1.38	1.45
9	0.93	0.86	1.40	1.33	1.40
10	1.01	0.93	1.51	1.44	1.52
11	1.07	0.99	1.60	1.53	1.61
12	1.21	1.12	1.81	1.73	1.82

Sleep \ WHR	0	1	2	3	4
3	0.09	-0.24	-0.13	0.05	0.13
4	0.05	-0.39	0.05	0.00	0.11
5	0.10	-0.21	0.11	-0.06	-0.02
6	0.10	-0.09	0.07	-0.01	-0.08
7	0.03	0.01	-0.00	-0.09	0.06
8	0.01	0.14	0.03	0.01	-0.15
9	0.08	0.06	-0.06	-0.05	0.01
10	0.04	0.02	-0.06	0.01	-0.00
11	-0.18	0.16	-0.04	0.01	0.03
12	-0.19	0.04	0.01	0.09	-0.01

Fig A: Sleep × WHR → Function under multiplicative independence. Left: multiplicative expectation on the log scale. Right: log-residual L_{ij}^{mult}; values above zero indicate observed/expected > 1.

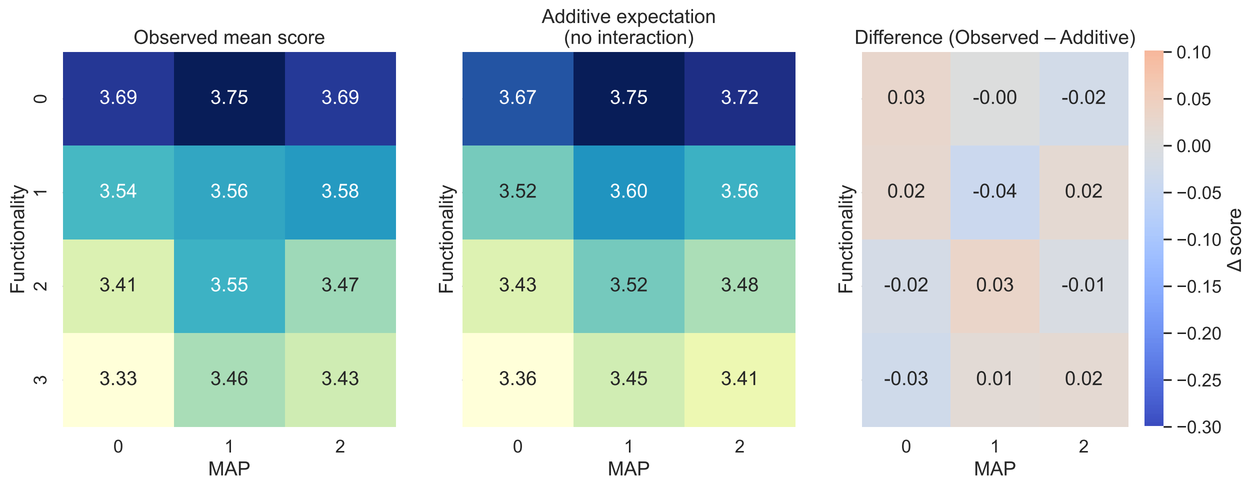

Fig B: Blood pressure (MAP) × Hearing → Function (non-synergistic). Left: observed means. Middle: additive expectation. Right: additive residuals showing minimal structure.

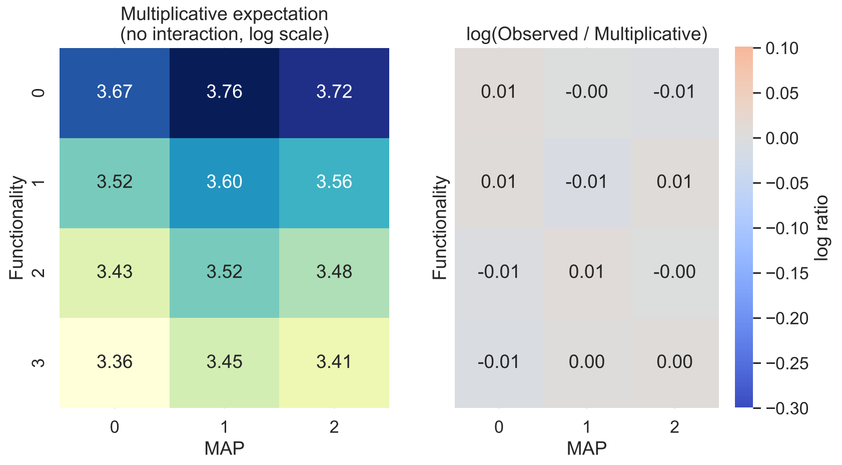

Fig C: MAP × Hearing → Function (non-synergistic), multiplicative view. Residuals on the log scale are near zero, consistent with negligible PID synergy.

S4. Rule-based joint-state patterns

To examine how specific constellations behaved with respect to individual outcomes, we evaluated the joint states of each constellation across all target variables not included among its members. All variables were discrete, and analyses were performed on the full population. For each constellation–target pair, we tabulated all observed joint states and computed the empirical mean of the target in each state. An ordinal regression model with the target as the response and the constellation variables as main-effect predictors served as an additive benchmark; expected values under this model were obtained by probability-weighted averaging across outcome categories.

Deviations were defined as the difference between the observed and additive-predicted means for each joint state. States with fewer than five observations were flagged as low-confidence. Rule-like configurations were those with absolute deviations in the upper 5% for that constellation–target combination. To assess whether these configurations contributed information beyond the main effects, we augmented the ordinal model with binary indicators for the flagged states and compared fit to the baseline model using the Akaike Information Criterion (AIC).

Across constellations, the number and magnitude of rule-like configurations varied. The Alcohol Use–grip strength pair typically produced a single rule-like state per target, indicating a focused interaction that nonetheless improved model fit for several outcomes (Table C). In contrast, the four-variable constellation comprising General Health, Pain, grip strength and Cognition Spell showed multiple rule-like states for most targets, with substantial improvements in AIC for behavioural, sensory, cognitive and functional measures (Table D). The Health-vs-Peers–Physical Activity–Sleep constellation showed an intermediate pattern with several rule-like states for selected outcomes (Table E), and the Sleep–Physical Activity pair exhibited a small number of rule-like states that improved fit particularly for pain, grip strength and functional status (Table F). Together, these findings show that synergistic constellations differ not only in the breadth and robustness captured by BUST, but also in the complexity of the joint configurations through which their non-additive effects emerge.

Table C: Summary of joint-state deviations and AIC comparison for the Alcohol Use–grip strength constellation across all target variables. For each target, the table reports the number of observed joint states (n_{states}), the number of rule-like configurations (n_{rules}), the maximum absolute deviation between observed and additive-predicted means ($\max|\Delta|$), the direction of this deviation, and the AIC values for the baseline and rule-augmented ordinal models.

Target	n_{states}	n_{rules}	$\max|\Delta|$	Direction	AIC (baseline)	AIC (rule)	ΔAIC
Anxiety	12	1	0.040	positive	15915.476	15914.230	-1.247
Depression	12	1	0.037	positive	7998.854	7967.211	-31.644
Sleep	12	1	0.250	positive	52727.930	52720.505	-7.425
Cognition	12	1	0.049	negative	9778.249	9733.655	-44.594
Pain	12	1	0.164	negative	32288.510	32266.560	-21.951
Hearing	12	1	0.059	negative	24295.210	24295.126	-0.084
General_Health	12	1	0.088	negative	22360.130	22329.158	-30.972
Health_vs_Peers	12	1	0.066	positive	27455.668	27444.609	-11.059
Physical_Activity	12	1	0.161	positive	26829.965	26781.926	-48.039
BMI	12	1	0.055	negative	29837.861	29834.426	-3.435
Memory_Problems	12	1	0.052	negative	18048.117	18043.363	-4.755
Memory_Complaints	12	1	0.052	negative	18048.117	18043.363	-4.755
WHR_Obesity	12	1	0.013	negative	10365.228	10365.768	0.540
Blood pressure_vs_H	12	1	0.035	negative	13981.366	13980.399	-0.967
functionality_state	12	1	0.191	negative	23665.826	23608.380	-57.446

Table D: Summary of joint-state deviations and AIC comparison for the General Health–Pain–grip strength–Cognition constellation across all target variables. For each target, the table reports the number of observed joint states (n_{states}), the number of rule-like configurations (n_{rules}), the maximum absolute deviation between observed and additive-predicted means ($\max|\Delta|$), the direction of this deviation, and the AIC values for the baseline and rule-augmented ordinal models.

Target	n_{states}	n_{rules}	$\max|\Delta|$	Direction	AIC (baseline)	AIC (rule)	ΔAIC
Anxiety	120	6	0.321	negative	14759.088	14747.285	-11.802
Depression	120	6	0.347	positive	6989.281	6973.826	-15.455
Sleep	120	6	1.110	negative	51736.208	51726.656	-9.553
Hearing	120	6	0.634	negative	24170.489	24158.966	-11.522
Health_vs_Peers	120	6	0.445	positive	24426.470	24409.246	-17.224
General_Health	120	6	0.285	positive	26509.280	26483.222	-26.058
BMI	120	6	0.223	negative	29633.362	29613.307	-20.056
Alcohol_Use	120	6	0.365	positive	28954.214	28928.156	-26.058
Memory_Problems	120	6	0.389	negative	17898.648	17878.392	-20.256
Memory_Complaints	120	6	0.389	negative	17898.648	17878.392	-20.256
grip_category	120	6	0.531	negative	36287.994	36257.636	-30.358
WHR_Obesity	120	6	0.139	negative	10402.478	10398.841	-3.637
Blood pressure	120	6	0.124	negative	14072.473	14069.857	-2.616
functionality_state	120	6	0.641	negative	20818.320	20798.862	-19.458

Table E: Summary of joint-state deviations and AIC comparison for the Health-vs-Peers–Physical Activity–Sleep constellation across all target variables. For each target, the table reports the number of observed joint states (n_{states}), the number of rule-like configurations (n_{rules}), the maximum absolute deviation between observed and additive-predicted means ($\max|\Delta|$), the direction of this deviation, and the AIC values for the baseline and rule-augmented ordinal models.

Target	n_{states}	n_{rules}	$\max\lvert\Delta\rvert$	Direction	AIC (baseline)	AIC (rule)	ΔAIC
Anxiety	63	4	0.105	negative	14621.916	14617.814	-4.101
Depression	63	4	0.133	positive	7130.283	7123.223	-7.060
Cognition	63	4	0.169	positive	9436.955	9419.692	-17.263
Pain	63	4	0.499	positive	29859.282	29838.100	-21.181
Hearing	63	4	0.526	negative	24187.219	24159.503	-27.716
General_Health	63	4	0.113	positive	21187.059	21180.274	-6.785
BMI	63	4	0.122	negative	29642.914	29630.769	-12.145
Alcohol_Use	63	4	0.213	positive	28971.610	28953.016	-18.594
Memory_Problems	63	4	0.263	negative	17911.274	17901.771	-9.503
Memory_Complaints	63	4	0.263	negative	17911.274	17901.771	-9.503
grip_category	63	4	0.536	negative	36382.812	36339.400	-43.412
WHR_Obesity	63	4	0.084	negative	10402.737	10399.146	-3.590
Blood pressure	63	4	0.049	negative	14073.040	14071.042	-1.997
functionality_state	63	4	0.265	negative	20817.028	20808.873	-8.155

Table F: Summary of joint-state deviations and AIC comparison for the Sleep–Physical Activity constellation across all target variables. For each target, the table reports the number of observed joint states (n_{states}), the number of rule-like configurations (n_{rules}), the maximum absolute deviation between observed and additive-predicted means ($\max|\Delta|$), the direction of this maximum deviation, and the AIC values for the baseline and rule-augmented ordinal models.

Target	n_{states}	n_{rules}	$\max\lvert\Delta\rvert$	Direction	AIC (baseline)	AIC (rule)	ΔAIC
Anxiety	21	2	0.054	positive	14788.512	14785.504	-3.008
Depression	21	2	0.049	negative	7273.374	7271.163	-2.211
Cognition	21	2	0.046	positive	9443.915	9440.324	-3.591
Pain	21	2	0.198	positive	30714.896	30712.994	-1.903
Hearing	21	2	0.167	negative	24189.537	24185.939	-3.598
General_Health	21	2	0.069	positive	21199.229	21197.192	-2.037
Health_vs_Peers	21	2	0.064	positive	27182.606	27183.270	0.664
BMI	21	2	0.069	negative	29646.440	29643.477	-2.963
Alcohol_Use	21	2	0.115	positive	28998.317	28971.297	-27.020
Memory_Problems	21	2	0.108	negative	17921.059	17905.550	-15.509
Memory_Complaints	21	2	0.108	negative	17921.059	17905.550	-15.509
grip_category	21	2	0.261	negative	36105.356	36076.138	-29.217
WHR_Obesity	21	2	0.024	negative	10404.314	10405.087	0.773
Blood pressure	21	2	0.036	negative	14074.111	14067.868	-6.243
functionality_state	21	2	0.089	negative	20841.871	20840.387	-1.483

S5. Simulation validation of the estimation pipeline

This section presents the full results of the three simulation experiments summarised in the Simulation validation of the estimation pipeline section of the main paper.

Cardinality sweep. Fig D shows synergy RMSE as a function of variable cardinality ($K = 2$–5) for each of the four target functions (modular copy, XOR, minimum, and redundancy-dominated) under $I_{\min}$. RMSE increases with K for all distributions except the redundancy-dominated function, whose true synergy is zero regardless of cardinality and is recovered with negligible error. Fig E shows the interaction between cardinality and sample size: at $K \leq 4$, synergy RMSE remains below 0.02 bits across all tested sample sizes ($N = 500$–$2{,}636$); at $K = 5$ with $N = 500$, RMSE rises to approximately 0.04 bits for the minimum function, the most challenging distribution tested.

LASA-matched cardinality profiles. Fig F reports RMSE for all four PID atoms across four LASA-matched cardinality profiles ($3 \times 4 \to 5$, $2 \times 2 \to 7$, $3 \times 3 \to 3$, $5 \times 7 \to 5$) under three information regimes (function-of-one, mixed, and synergy-rich). All atoms are recovered with RMSE < 0.02 bits at the largest tested sample size for most profile–regime combinations. The highest-cardinality profile ($5 \times 7 \to 5$) exhibits moderately elevated RMSE for the synergy atom under the synergy-rich regime (≈ 0.08 bits), consistent with the cardinality sweep results.

BUST score recovery. Fig G shows estimated vs. true BUST scores (Breadth, Synergy strength, Total information) for three synthetic source pairs with distinct breadth profiles. Synergy strength and Total information fall close to the identity line across all pairs and sample sizes. Breadth exhibits a systematic downward bias for the broad-synergy pair (true $B \approx 0.96$, estimated $B \approx 0.78$), consistent with estimation noise deflating normalised entropy; the narrow-synergy pair ($B \approx 0$) is recovered accurately. The relative ranking of pairs by all three BUST components is preserved across all conditions.

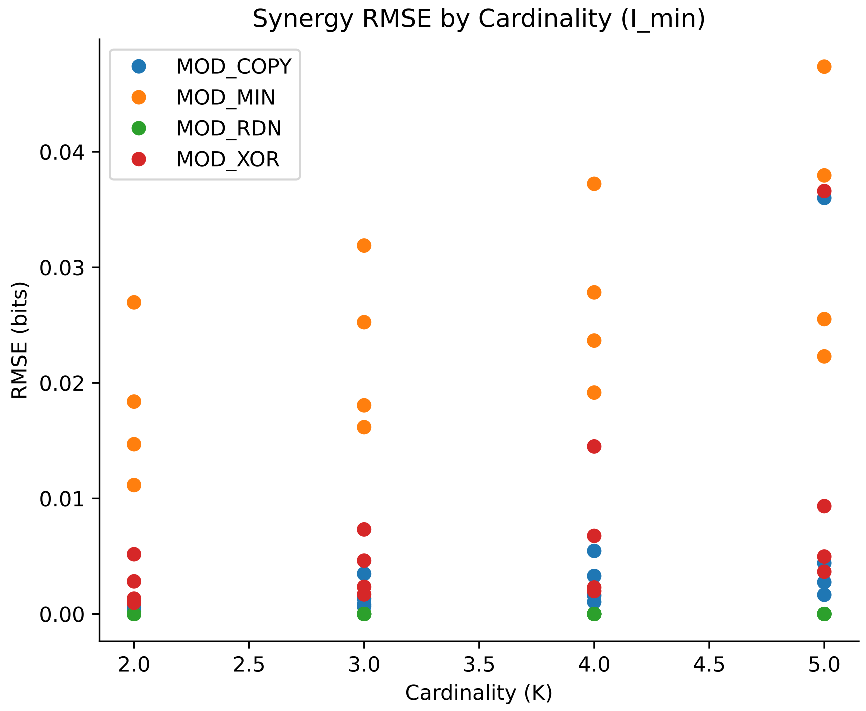

Fig D: Synergy RMSE as a function of variable cardinality (K) for four deterministic target functions under $I_{\min}$, pooled across sample sizes and source-coupling conditions. Each point represents one condition (sample size $\times$ coupling); vertical spread shows variation across these conditions.

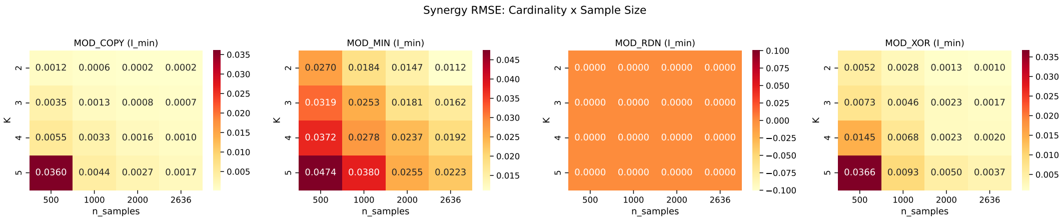

Fig E: Interaction between variable cardinality and sample size on synergy RMSE ($I_{\min}$, independent sources). Each cell shows the RMSE (bits) for one distribution–sample-size combination.

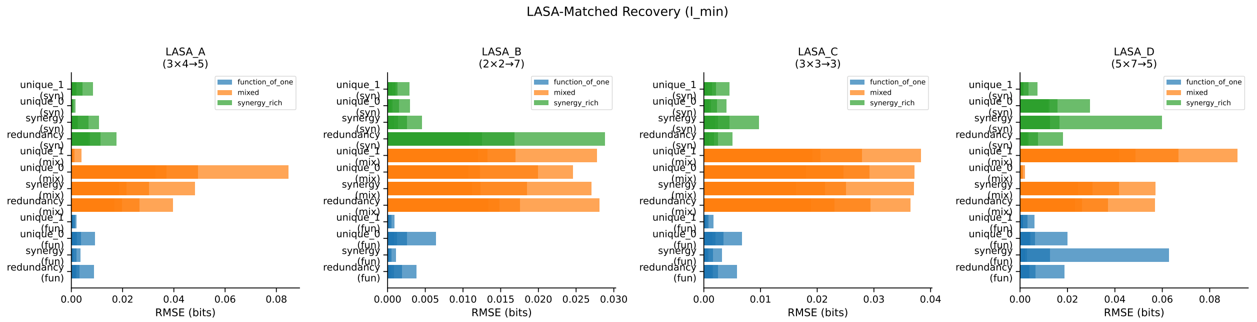

Fig F: PID atom recovery under four LASA-matched cardinality profiles and three information regimes ($I_{\min}$, $N = 2{,}636$). Horizontal bars show RMSE (bits) for each atom–regime combination. Lower-cardinality profiles (LASA_B: $2 \times 2 \to 7$; LASA_C: $3 \times 3 \to 3$) achieve the lowest RMSE; the highest-cardinality profile (LASA_D: $5 \times 7 \to 5$) shows moderately elevated RMSE for synergy under the synergy-rich regime.

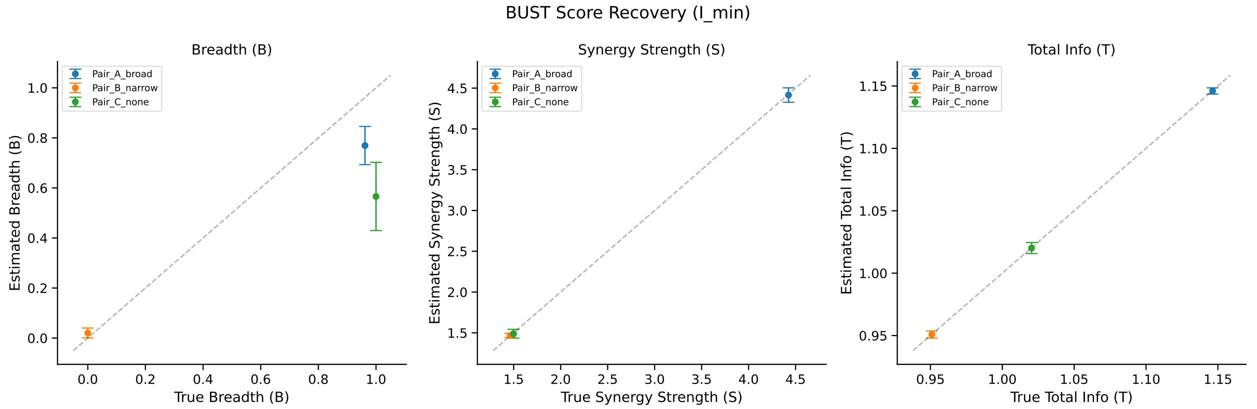

Fig G: BUST score recovery: estimated vs. true values for Breadth (B), Synergy strength (S), and Total information (T) across three synthetic source pairs with known breadth profiles (broad, narrow, none). Points show means across replicates; error bars show ±1 SD. The dashed line is the identity. Synergy strength and Total information are recovered accurately; Breadth shows a conservative downward bias for the broad-synergy pair.

S6. Sensitivity to discretisation choices

To assess the robustness of BUST scores to discretisation, we re-ran the full PID and BUST pipeline under five alternative binning strategies applied to all continuous and ordinal variables with more than two levels:

1. **Clinical** (primary analysis): cut-points based on clinical guidelines and LASA documentation, with post-hoc sparse-state merging (see PID estimation section of the main paper).
2. **Equal-frequency tertiles**: each variable split into three bins of approximately equal size.
3. **Equal-frequency quartiles**: each variable split into four equal-frequency bins.
4. **Equal-width terciles**: each variable split into three bins of equal range.
5. **Coarsened binary**: each variable dichotomised at its median.

Binary variables (e.g., memory complaints, anxiety, WHR obesity) retained their original coding under all schemes. For each scheme, PID atoms were estimated with QE bias correction and target-shuffle baseline subtraction, and BUST composite scores were computed identically to the primary analysis.

Rank correlations. Table G reports pairwise Spearman rank correlations of composite BUST scores across schemes. Among the four non-binary schemes, correlations range from 0.83 to 0.96, indicating that the relative ordering of source pairs by BUST score is largely preserved regardless of whether clinical, equal-frequency, or equal-width bins are used. The equal-frequency tertile and quartile schemes are nearly interchangeable ($\rho = 0.96$). The coarsened binary scheme is poorly correlated with all others ($\rho = 0.06$–0.18; all $p > 0.1$), reflecting the severe information loss when multi-level variables are collapsed to two categories.

Table G: Pairwise Spearman rank correlations of composite BUST scores across five discretisation schemes. Bold values indicate $p < 0.001$.

	Clinical	EF-3	EF-4	EW-3	Binary
Clinical	1.00	**0.84**	**0.83**	**0.85**	0.18
EF-3		1.00	**0.96**	**0.90**	0.06
EF-4			1.00	**0.86**	0.14
EW-3				1.00	0.10
Binary					1.00

Top-pair overlap. The Jaccard index for the top-10 ranked pairs between equal-frequency tertiles and quartiles was 0.82, confirming that the highest-ranked BUST edges are robust to granularity within equal-frequency schemes. Between clinical and equal-frequency schemes, top-10 Jaccard indices ranged from 0.18 to 0.25, reflecting genuine differences in how clinical cut-points versus data-driven quantiles partition variable space. The source pair Physical Activity–Memory Complaints appeared in the top 10 under all four non-binary schemes. The binary scheme shared no top-10 pairs with the clinical scheme (Jaccard = 0.0).

BUST quadrant stability. Pairwise BUST quadrant agreement (broad vs. narrow classification) among the four non-binary schemes ranged from 59% to 84% (mean 72%). Including the binary scheme reduced overall agreement to 27%, driven by the binary scheme's tendency to assign high breadth scores to all pairs owing to the collapse of multi-level variables. The poor agreement of the coarsened binary scheme is expected: by the data processing inequality (Cover & Thomas, *Elements of Information Theory*, 2005), collapsing multi-level variables to two categories can only reduce mutual information, and the resulting $2 \times 2 \times 2$ joint triplet states provide far less resolution for non-additive patterns to manifest than the richer joint state spaces available under multi-level discretisations.

Visual comparison of BUST dimensions. Because each binning scheme was applied to the full population only (without stratification), the Uniformity dimension cannot be estimated and is omitted from the visual comparison. Fig H displays the three remaining BUST dimensions - Breadth (B), Synergy strength (S), and Total information (T) - for each scheme, using an encoding that mirrors the main BUST map (Fig 2): the x-axis shows median-centred Breadth, the y-axis shows Synergy strength in bits, point size encodes Total information, and point colour encodes the Synergy fraction (S/T). The four non-binary schemes produce visually similar point clouds, with the same pairs occupying high-synergy, moderate-breadth positions across panels. The coarsened binary scheme shows a markedly different structure: most pairs are pushed to high breadth (because binary variables yield near-uniform synergy shares across targets) and low synergy strength, consistent with the information loss from collapsing multi-level variables.

Fig I summarises the pairwise Spearman rank correlations of composite BUST scores, and Fig J shows the Jaccard overlap of the top-10 ranked pairs for each scheme comparison. Fig K provides an alternative view of the rank correlation structure.

S7. Bootstrap confidence intervals for synergy estimates

To assess the sampling variability of synergy estimates for the highest-ranked BUST pairs, we performed a nonparametric bootstrap with 1,000 replicates. In each replicate, the full-population discretised dataset (N = 14,716 observations) was resampled with replacement, and QE-corrected PID atoms were re-estimated for the 148 (pair, target) combinations associated with the top-10 composite BUST pairs. Percentile-based 95% confidence intervals were computed for each combination and for the per-pair mean synergy across targets.

Fig L shows the resulting CIs for per-pair mean synergy, ordered by point estimate. All ten pairs have CIs that exclude zero. The fraction of individual targets whose synergy CI excludes zero ranges from 73% (memory complaints–physical activity; memory problems–physical activity) to 93% (physical activity–sleep; pain–grip strength), indicating that the synergistic signal is distributed across most targets rather than driven by isolated cases.

Fig M presents target-specific synergy CIs for the top-5 pairs, showing which targets contribute most to each pair's aggregate synergy. For example, the pain–physical activity pair derives its strongest synergy from functional limitations ($\approx$ 0.12 bits), whereas its synergy with respect to most other targets is an order of magnitude smaller.

Fig N characterises the precision of individual estimates. The median CI width across all 148 (pair, target) combinations was 0.005 bits, and CI width scaled approximately linearly with effect size, indicating that precision is adequate across the range of observed synergies.

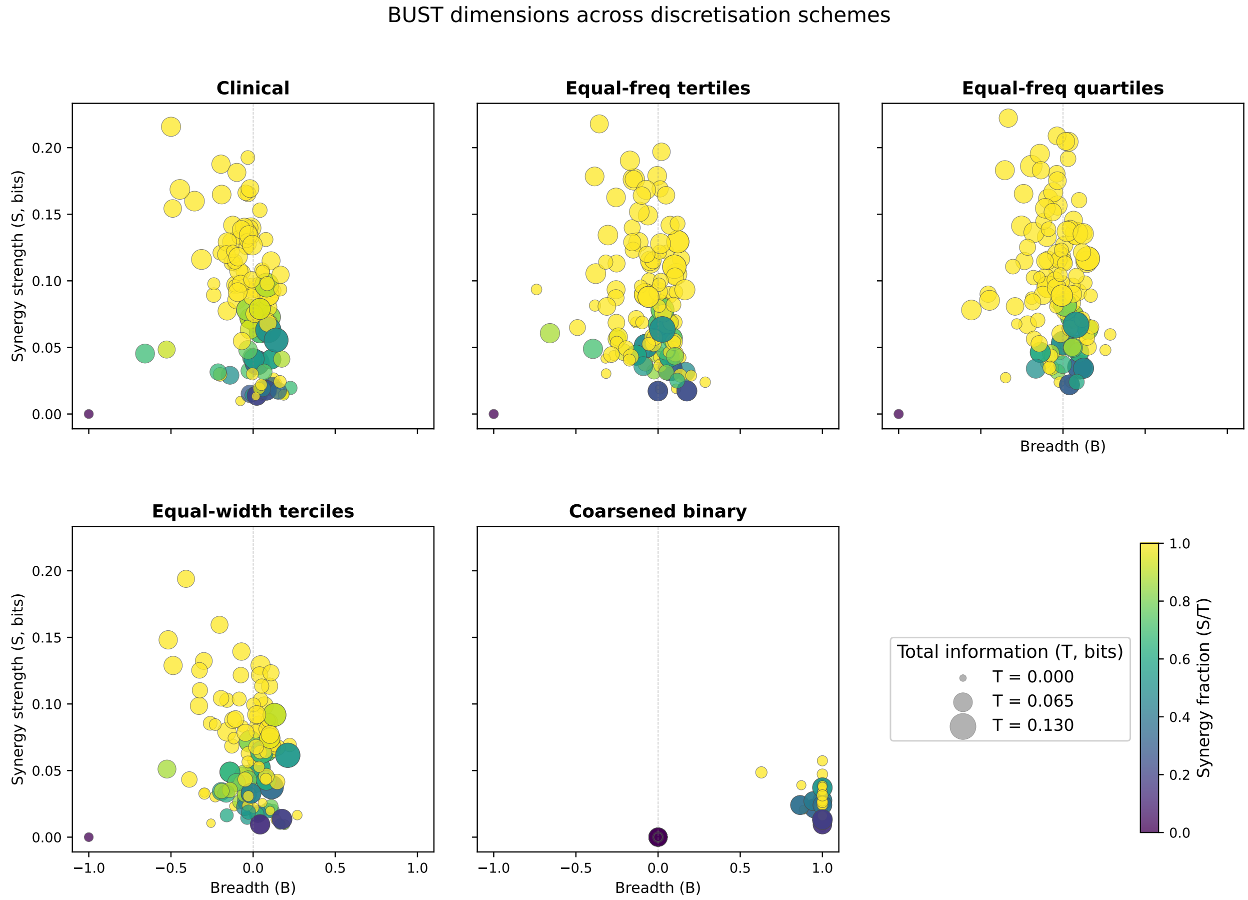

Fig H: Breadth–Synergy scatter for all source pairs under five discretisation schemes. The x-axis shows median-centred Breadth (B); the y-axis shows Synergy strength (S, bits); point size encodes Total information (T, bits); point colour encodes Synergy fraction (S/T). The Uniformity dimension is omitted because each scheme was evaluated on the full population only. The four non-binary schemes produce similar point clouds, whereas the coarsened binary scheme shows a distinctly different pattern with compressed synergy magnitudes and inflated breadth.

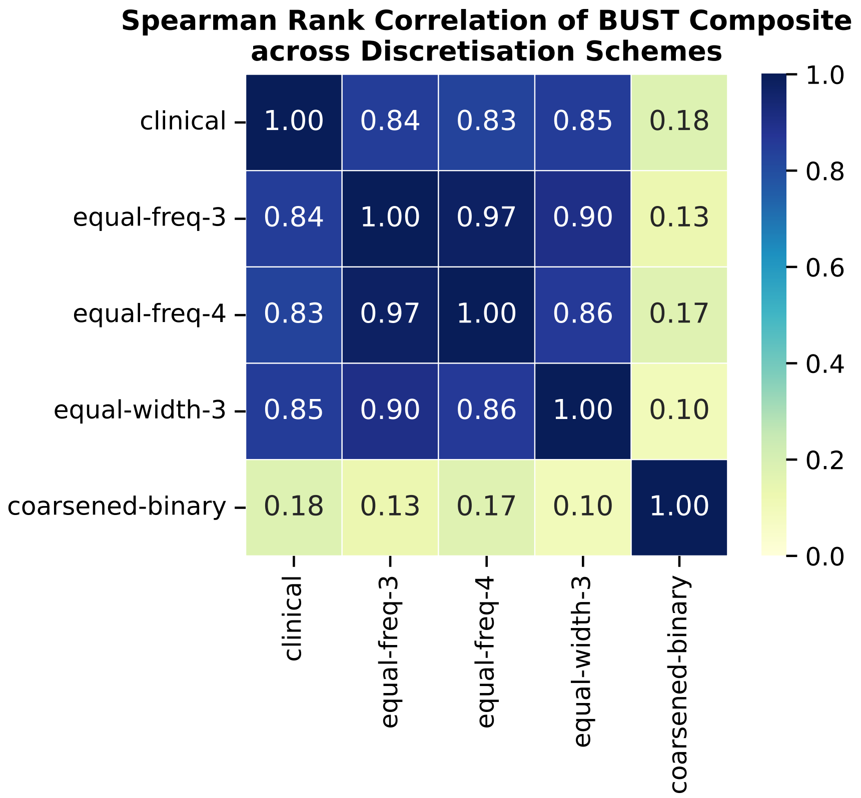

Fig I: Spearman rank correlation matrix of composite BUST scores across five discretisation schemes. Non-binary schemes are highly correlated ($\rho = 0.83$–0.96), while the coarsened binary scheme is poorly correlated with all others ($\rho = 0.06$–0.18).

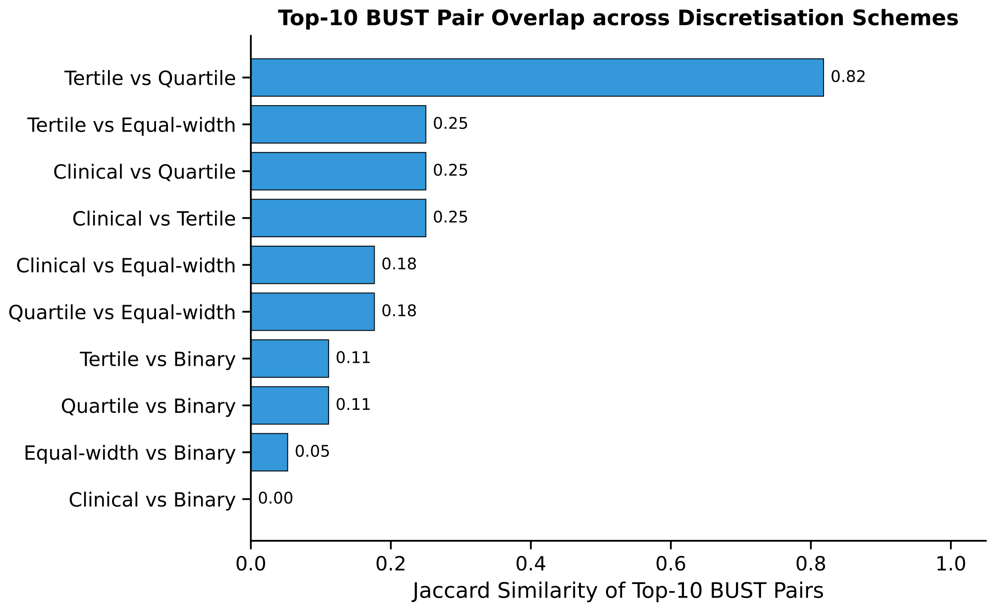

Fig J: Jaccard similarity of the top-10 ranked BUST pairs across all pairwise scheme comparisons. The equal-frequency tertile and quartile schemes share 82% of their top-10 pairs. Comparisons involving the coarsened binary scheme show near-zero overlap.

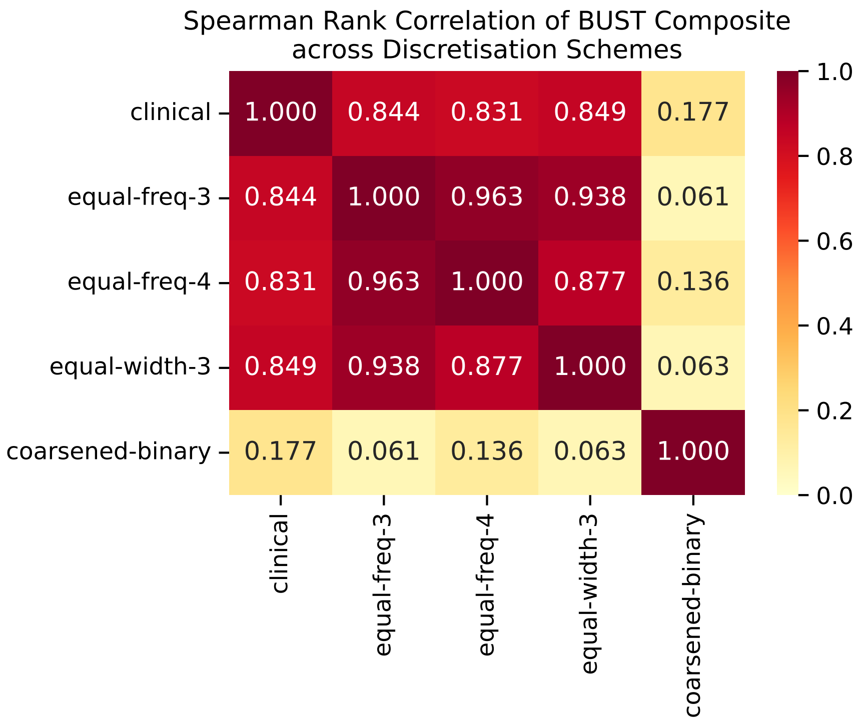

Fig K: Spearman rank correlations of composite BUST scores between each pair of discretisation schemes, displayed as an annotated heatmap.

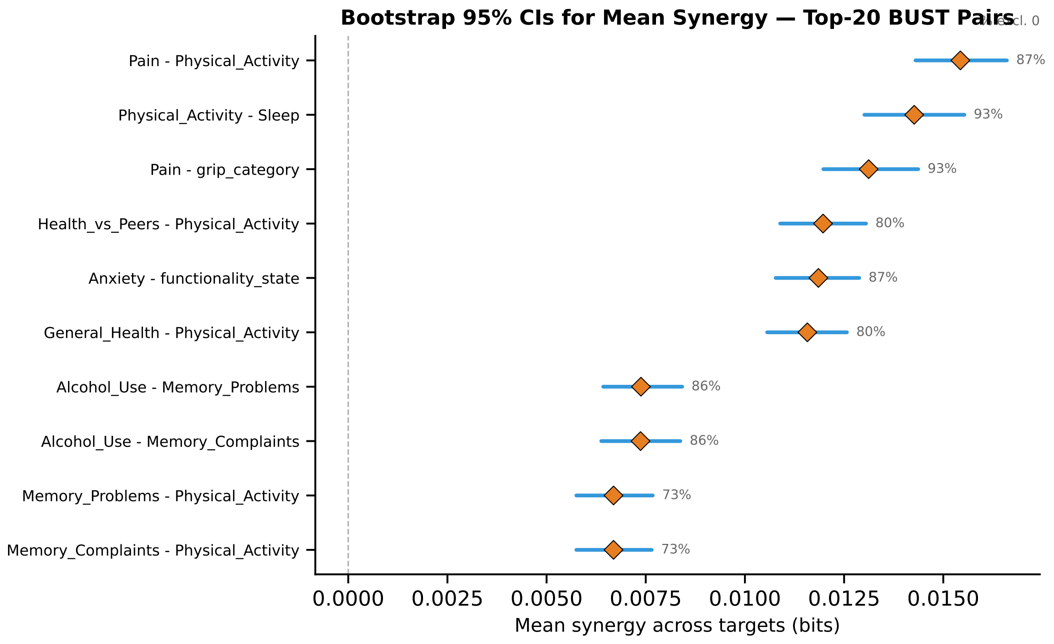

Fig L: **Bootstrap 95% confidence intervals for mean synergy across targets for the top-10 BUST pairs.** Diamond markers show the point estimate (mean synergy across all targets); horizontal bars show percentile-based 95% CIs from 1,000 bootstrap replicates. The percentage to the right of each bar indicates the fraction of individual targets whose target-specific synergy CI excludes zero. All ten pairs have aggregate CIs that exclude zero.

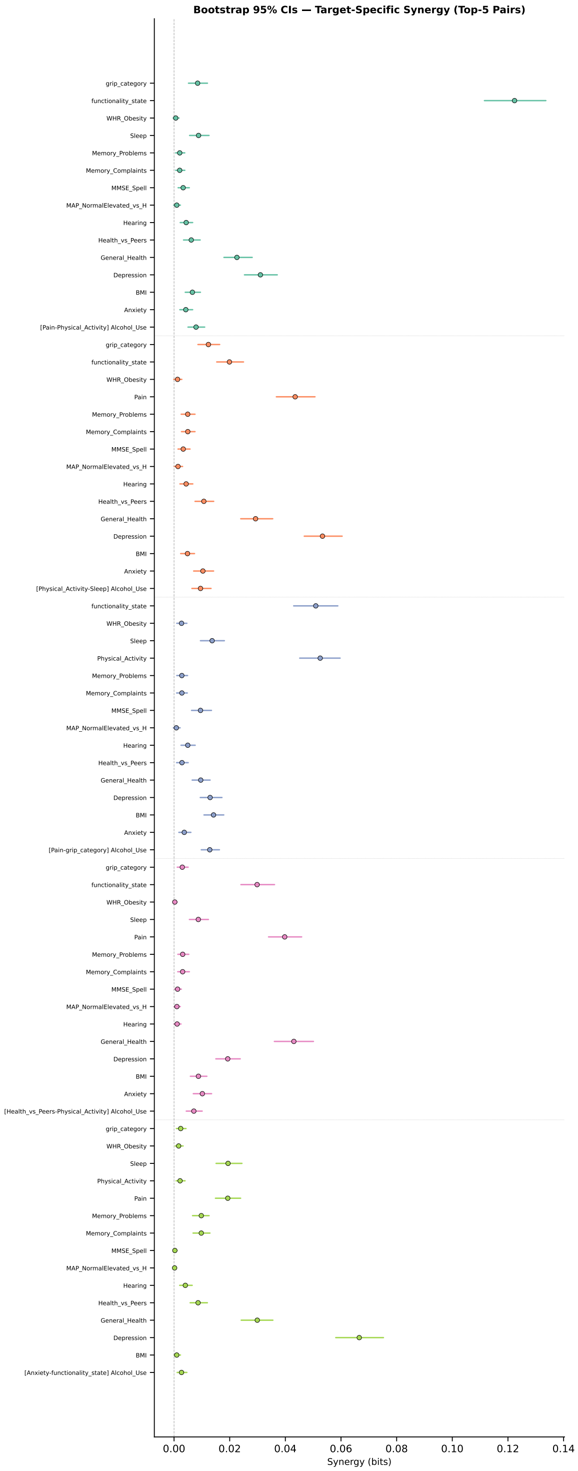

Fig M: **Target-specific bootstrap 95% CIs for synergy in the top-5 BUST pairs.** Each panel corresponds to one source pair; rows within panels show individual targets. Horizontal bars are percentile-based 95% CIs (1,000 replicates). The variation in synergy magnitude across targets illustrates the breadth dimension of the BUST representation.

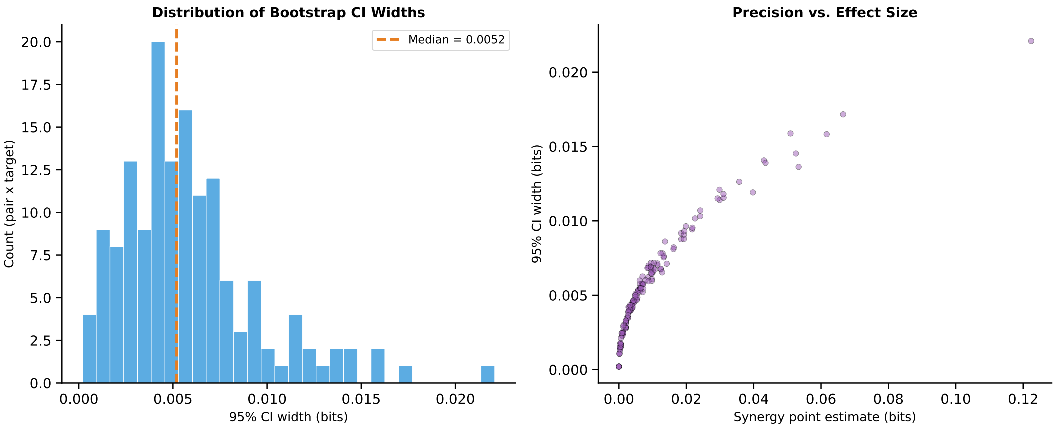

Fig N: **Distribution of bootstrap CI widths.** Left: histogram of 95% CI widths across all 148 (pair, target) combinations (median = 0.005 bits). Right: CI width plotted against the synergy point estimate, showing an approximately linear relationship between precision and effect size.
