# Supplementary figures and images for: Interpreting higher-order dependence in multimorbidity using cohort data: A partial information decomposition approach
Source: PLoS Comput Biol. 2026 Jun 10;22(6):e1014386. doi: 10.1371/journal.pcbi.1014386 (PMC13268148; doi:10.1371/journal.pcbi.1014386)

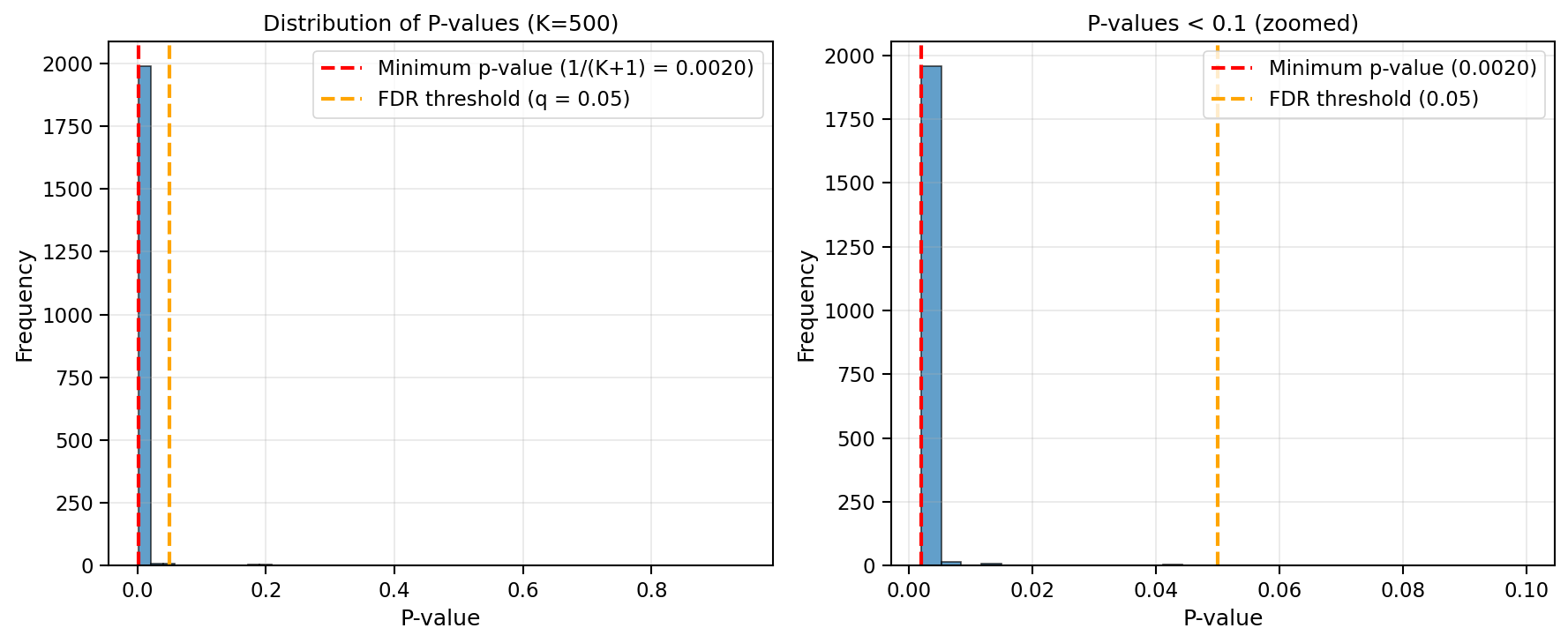

Supplement: S1 File — PID_BUST_Results_and_DemoScripts.zip is a self-contained archive enabling readers to reproduce the main-text analyses and figures from pre-computed PID results. It contains a Jupyter notebook that regenerates Figs 1–4, plotting utility modules, pre-computed permutation-screening results and analysis-ready dataframes, BUST-network artefacts (edge lists, node attributes, clique catalogues, and quadrant assignments), hub-variable summaries, and a Python requirements file. Installation and execution are documented in the accompanying README. Raw LASA participant data are not included (governed access); the package provides the full downstream pipeline conditional on data access. (ZIP) [file pcbi.1014386.s002.zip › PID_BUST_Results_and_DemoScripts/screening_results_500perms_v21/permutation_diagnostics.png]
